# Breaking the Thick Electrode Paradox With an in situ VS_2_@V_2_CTx MXene Heterostructure for High‐Areal‐Capacity Batteries

**DOI:** 10.1002/advs.202522300

**Published:** 2025-12-21

**Authors:** Lirong Wang, Jiulong Li, Ye Chen, Cheng Zhang, Youquan Jiang, Chaoyu Chen, Haonan Song, Peigen Zhang, Sheng Liu, Zhaodong Li

**Affiliations:** ^1^ School of Integrated Circuits Wuhan University Wuhan China; ^2^ School of Materials Science and Engineering Southeast University Nanjing China

**Keywords:** fast charge transport kinetics, high areal capacity, thick electrode paradox, VS2@V2CTx heterostructures

## Abstract

Achieving high areal capacity is critical for advancing lithium‐ion batteries (LIBs) toward high‐energy‐density applications. However, prevailing thick electrode architectures inevitably suffer from sluggish charge transport kinetics and mechanical degradation. Here, we pioneer an in situ gas‐phase conversion strategy to directly grow metallic VS_2_ nanosheets on V_2_CTx MXene within a multi‐walled carbon nanotube (MWCNT) network. This integrated architecture simultaneously establishes interpenetrating electron/ion highways—enabling full lithiation in ultra‐thick electrodes (12‐fold higher Li^+^ concentration compare to the traditional electrode)—and enhances mechanical toughness, thus achieving exceptional cycling stability under high current densities. Such TMDC–MXene heterostructure exhibit an impressive specific capacity of 1046 mAh g^−1^ and areal capacity of 13.6 mAh cm^−2^, with the thickness of 300 µm, representing only 12.8% gravimetric capacity decay despite 333% increased electrode thickness. Moreover, the resulting electrode maintains excellent cycling stability, retaining a capacity of 1.8 mAh cm^−2^ even at high current density of 6.4 mA cm^−2^, alongside 81% capacity retention over 600 cycles at 2C in LiFePO_4_ full‐cells.

## Graphical abstract

This work pioneers an in situ gas‐phase conversion strategy to construct VS_2_@V_2_Tx heterostructures within a MWCNT network. The integrated architecture establishes interpenetrating electron/ion highways, enabling an ultra‐thick electrode (300 µm) to achieve a high areal capacity of 13.6 mAh cm^−2^ with exceptional cycling stability, demonstrating great promise for high‐energy‐density batteries.

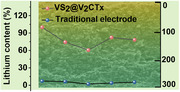

## 1 Introduction

Efforts to advance lithium‐ion batteries (LIBs) have historically prioritized specific capacity (mAh g^−1^), yet this mass‐based metric inadequately reflects system‐level energy density and manufacturability. Areal capacity density (mAh cm^−2^) has emerged as a more holistic figure of merit, directly linking electrode architecture to practical energy output [1]. By increasing active material loading while preserving charge transport, high‐areal‐capacity battery electrodes simultaneously enhance gravimetric and volumetric performance [2]. This paradigm shift is exemplified by silicon anodes, which, despite their high theoretical capacity (∼4,200 mAh g^−1^), suffer from volumetric expansion and interface degradation, limiting their practical utility [3, 4]. Moreover, to satisfy the ever‐growing demands for high energy density electrical vehicles and large‐scale energy storage systems, thick electrode has been proposed and proven to be an effective way to achieve high energy density, but simultaneously hindered by inferior mechanical stabilities, poor charge transport kinetics, severe lithiation heterogeneities and anabatic thermal risks, underscoring the “thick electrode paradox” [1]. Recent breakthroughs—such as graded porosity architectures, elastic binders, and dry electrode processing—circumvent these challenges, enabling stable areal capacities >6 mAh cm^−2^ and elevating full‐cell energy density by over 30% without altering chemistry [5]. Notably, commercial advances like Tesla's 4680 cell and GAC's all‐solid‐state battery highlight how electrode architecture, rather than material substitution, underpins next‐generation performance [6]. As emerging fabrication methods such as micro‐gravure printing and solvent‐free coating unlock high‐loading precision at scale, areal capacity is positioned as a central design axis—aligning electrochemical innovation with industrial scalability and accelerating progress toward the 500 Wh kg^−1^ targets envisioned for 2030 [7].

Theoretically, achieving high areal capacity in LIB electrodes hinges on three critical imperatives to address the “thick electrode paradox” that arises from increasing active material loading in thicker electrodes: (1) enhancing intrinsic material capacity, (2) optimizing charge/ion transport through low‐tortuosity pathways [8], and (3) improving mechanical‐electrochemical coupling [9, 10, 11]. Besides, the solid electrolyte interphase (SEI) critically determines cycling stability by synergistically impacting all three of these factors: its chemical composition (e.g., inorganic‐organic hybrid vs. single‐component architectures) governs parasitic reaction resistance to preserve intrinsic capacity, while structural configuration (compact vs. porous morphologies) dictates lithium‐ion transport kinetics through low‐tortuosity pathways, and mechanical durability ensures robust mechanical‐electrochemical coupling at interfaces—positioning SEI as the cornerstone of advanced long‐cycle‐life battery [12, 13]. Recent progress in silicon‐based anodes exemplifies the first imperative, with Si–graphite composites (15%–25% Si) delivering ∼16% energy density enhancement in full cells, and graphene‐wrapped Si nanoparticles achieving 3.13 mAh cm^−2^ at 5 mg cm^−2^ loading—substantially outperforming pure graphene counterparts [14]. However, the electrode materials advances are constrained not only by their synthesis challenges but also the inherent instability and parasitic side reactions during lithiation, such as instable SEI formation—which induces progressive interfacial charge transfer resistance and accelerates capacity fade through irreversible lithium inventory loss, fundamentally limiting long‐term cyclability [15, 16]. Kinetics‐driven approaches, such as nanostructuring and heterostructure design, address charge transport and instable change accommodation but suffer from trade‐offs, including reduced Coulombic efficiency and weak interfacial adhesion in heterostructures, compromising long‐term cycling stability [17, 18, 19]. Composite electrodes offer flexibility but face challenges in maintaining mechanical integrity and minimizing dead volume at high mass loadings [18, 20]. Interfacial engineering, though promising in improving electrode stability, is hindered by poor adhesion between active materials and current collectors. While functionalized current collectors alleviate some of this, delamination remains a concern in thick electrodes. These limitations underscore the need for innovative electrode designs that integrate nanoengineering, interface chemistry, and mesoscale structural coherence. Emerging heterostructures provide a potential solution by synchronizing strain distribution and improving interface durability, offering a pathway to realizing high‐areal‐capacity anodes without sacrificing energy density, stability, or scalability.

The integration of metallic transition metal dichalcogenides (TMDCs) with MXene heterostructures offers a transformative platform to simultaneously enhance areal capacity density and mechanical durability in lithium‐ion batteries, driven by their synergistic electronic and structural attributes. Metallic TMDCs (e.g., 1T‐VS_2_, conductivity ∼10^6^ S cm^−1^) facilitate ultrafast electron transport, while MXenes exhibit exceptional Li⁺ intercalation kinetics due to their low diffusion barriers and tunable surface terminations [8, 21, 22, 23, 24]. Structural coherence is maintained as TMDCs form percolating conductive networks within the MXene matrix, and their expanded interlayer spacing synergizes with MXene's low‐tortuosity nanochannels to enable efficient ion transport at high mass loadings. The hybridization of TMDCs’ intercalation/conversion mechanisms with MXene's surface‐driven pseudocapacitance yields hierarchical charge storage across bulk and interfacial regions [8, 25, 26, 27, 28, 29]. Recent efforts emphasize interface engineering and spatial uniformity, employing techniques such as CVD and hybrid CVD–MBE to achieve high crystallinity, while hydrothermal and wet‐chemical methods offer cost‐effective routes to interfacial homogeneity [30, 31]. Nonetheless, scalability remains challenged by substrate limitations, energy demands, batch inconsistency, and issues such as MXene restacking and non‐uniform TMDCs deposition, which impede ion diffusion and degrade long‐term stability [32]. Overcoming these limitations requires atomic‐level control of heterointerface stoichiometry, robust mechanical coupling to current collectors, and scalable fabrication protocols. Ultimately, breakthroughs in areal capacity depend on multidimensional design strategies—combining interfacial optimization, hierarchical porosity, and active material‐current collectors‐compatible processing—to translate material‐level advances into industrially viable high‐performance electrodes.

Here, we demonstrate a gas‐phase conversion strategy to fabricate high‐areal‐capacity electrodes through in situ conversion of metallic VS_2_ nanosheets on V_2_CT_X_ MXene integrated with MWCNT current collectors. The in situ heterostructure enhances conductivity and interpenetrating electron/ion transport pathways, and improved electrode mechanical toughness, fully lithiation ensures long‐term mechanical and electrochemical stability under high current densities. As a result, the as‐synthesized VS_2_@V_2_CT_X_ electrode delivers an exceptional areal capacity of 13.6 mAh cm^−2^ at active material loading of 13 mg cm^−2^ with thicknesses of up to 300 µm, representing only 12.8% gravimetric capacity decay despite 333% increased electrode thickness, outperforming MXene‐based (e.g., phosphorene/Ti_3_C_2_TX ∼1 mAh cm^−2^ at 2 mg cm^−2^) [33], and TMDC composite electrodes (e.g., MoS_2_‐graphene ∼0.8 mAh cm^−2^ at 1.27 mg cm^−2^) [34]. Moreover, the electrode demonstrates exceptional cycling stability at high current densities, retaining areal capacities of >1.8 mAh cm^−2^ (6.4 mA cm^−2^) for 100 cycles (Si@Ti_3_C_2_Tx ∼ 1.12 mAh cm^−2^ at 0.5 mA cm^−2^ for 100 cycles, MoS_2_/GDYO ∼1.5 mAh cm^−2^ at 0.15 mA cm^−2^ for 100 cycles) [35, 36], effectively breaking the longstanding paradox between thick electrode and rapid charge/discharge kinetics, as well as the mechanical instability in conventional thick electrodes. Furthermore, in full‐cell configurations paired with LiFePO_4_ cathodes, the VS_2_@V_2_CT_X_ anode maintains cycling stability, retaining 81% capacity retention over 600 cycles at 2C. Simultaneously, the solvent‐free fabrication strategy supports environmentally friendly, scalable manufacturing without energy‐intensive slurry casting. This strategy establishes a scalable platform for engineering next‐generation high‐areal‐capacity batteries, synergizing high energy density with industry‐standard electrode manufacturability.

## 2 Results and Discussion

**FIGURE 1:**
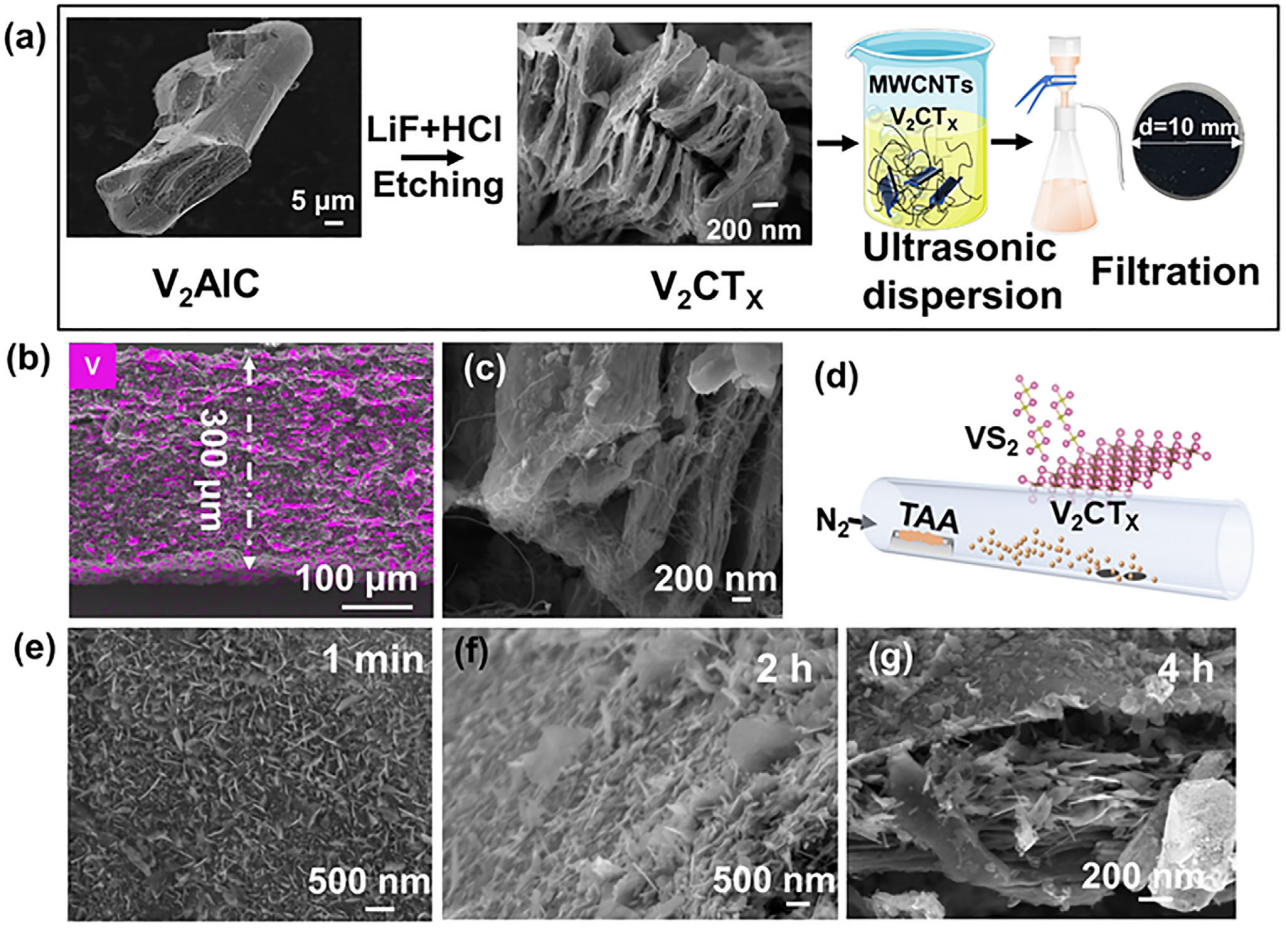
(a) Schematic illustration showing the V_2_CT_X_ and integrated with MWCNTs. (b) Cross‐sectional scanning electron microscopy (SEM) images of the integrated electrode of V_2_CT_X_ with MWCNTs (300x magnification). (c) MWCNTs are interspersed and wound around the opening of accordion like V_2_CT_X,_ (d) Schematic diagram of in situ gas‐phase conversion. (e–g) SEM images showing the morphological evolution of VS_2_@V_2_CT_X_ with increasing sulfurization time at 700°C: (e) 1 min, (f) 2 h, and (g) 4 h.

**FIGURE 2:**
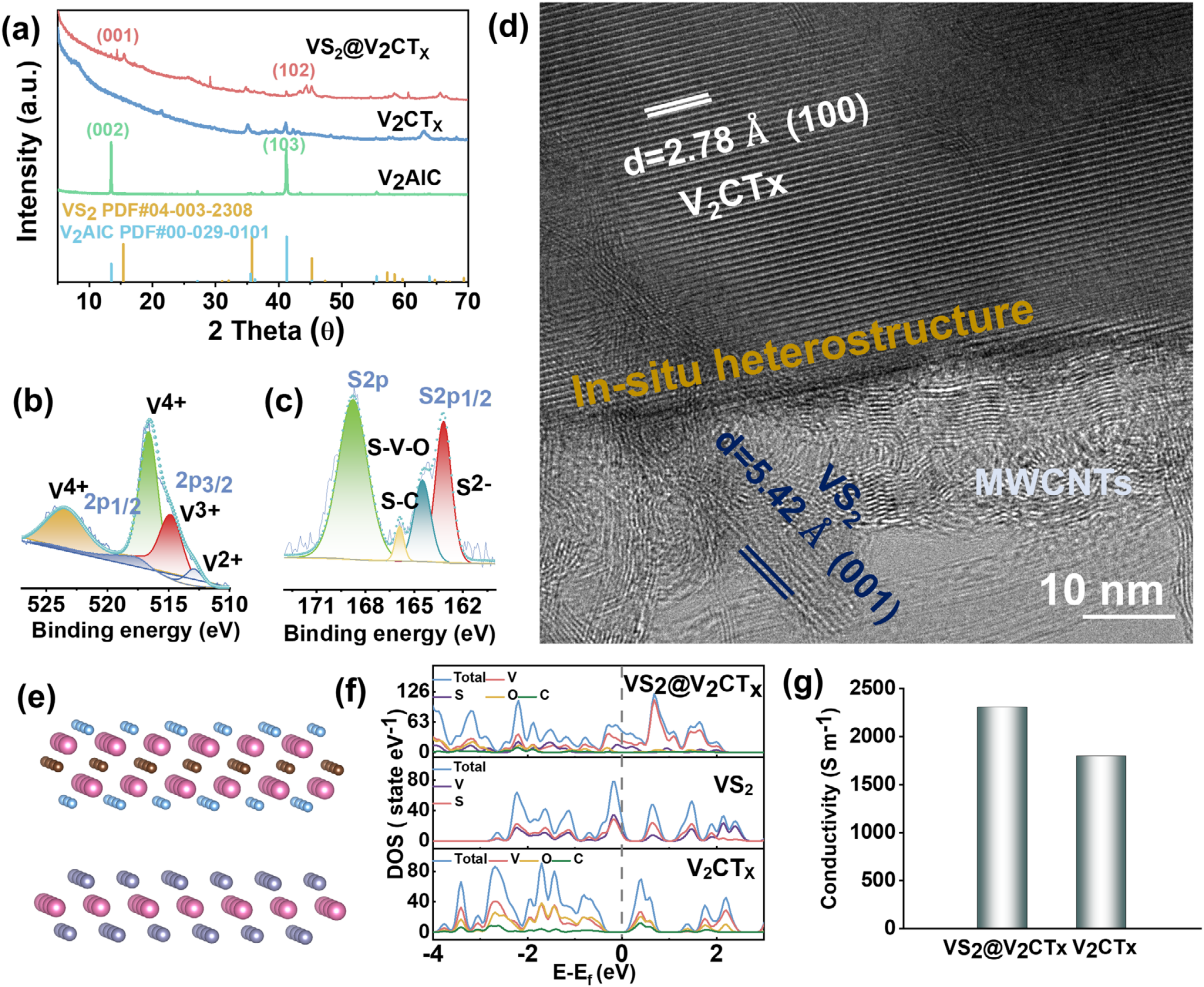
(a) XRD patterns of VS_2_@V_2_CTx, V_2_CTx and V_2_AlC. High‐resolution XPS spectra of the VS_2_@V_2_CTx sample: (b) V 2p and (c) S 2p regions. (d) HRTEM image showing the in situ heterostructure of VS_2_@V_2_CTx. (e) Optimized configuration of VS_2_@V_2_CT_X_ heterostructure. (f) DOS for VS_2_@V_2_CT_X_, V_2_CT_X_ MXene and VS_2_ models. (g) Conductivity of VS_2_@V_2_CTx and V_2_CTx electrodes by with four points probe method (take the average of three measurements).

We report a rationally designed, hierarchical VS_2_@V_2_CTx heterostructure anode synthesized via an in situ gas‐phase conversion strategy, achieving a synergistic integration of high areal capacity, cycle stability, and rate capability. As schematically illustrated in Figure 1a, bulk V_2_AlC powders are chemically etched into accordion like V_2_CT_X_, the structural transformation from V_2_AlC to V_2_CTx is confirmed by the downshift and broadening of the (002) and (103) XRD peaks (Figure 2a). V_2_CTx were subsequently integrated with multi‐wall carbon nanotubes (MWCNTs) to form a freestanding composite membrane, cut into a disc with a diameter of 10 mm with the thickness of up to 300 µm for use as negative electrode of Li‐ion battery (Figure 1b; Figure S1). The conductive MWCNTs not only serve as a lightweight current collector but also confine the electroactive V_2_CT_X_ within a 3D conductive scaffold, enabling homogeneous electron/ion transport and mitigating mechanical degradation under high mass loading (Figure 1c). The in situ gas‐phase conversion step is central to synthetic strategy (Figure 1d). Under a nitrogen atmosphere, thermally activated thioacetamide (TAA) vapor reacts with V_2_CT_X_ to grow vertically aligned VS_2_ nanosheets on the MXene surface. When sulfurized at 700 °C for 1 min, initial VS_2_ formation is observed with the prolongation of time, with the emergence of the (001) diffraction peak of VS_2_ gradually becomes apparent and sharp (Figure 1e–g; Figure S2). Prolonged reaction (up to 4 h) yields hexagonal VS_2_ nanosheets with enhanced crystallinity and a well‐defined morphology. Benefiting from the porous integrated structure of V_2_CT_X_ and MWCNTs, the gas‐phase reaction ensures a uniform sulfur infusion and subsequent VS_2_ formation deep within the electrode, leading to a homogeneous distribution of active materials (Figure S3). The growth of VS_2_ is evidenced by the sharp (001) VS_2_ peak and the presence of V^4+^ and S^2−^ species in XPS spectra (Figure 2b,c). High‐resolution TEM and selected area electron diffraction (SAED) reveal uniform, single‐crystalline VS_2_ nanosheets strongly anchored to the V_2_CT_X_ matrix (Figure S4a–h). In addition, Figure 2d clearly shows the in situ interface between VS_2_ and V_2_CTx. The interpenetrating dual‐phase network ensures efficient electron and ion transport, forming robust interfacial charge transfer pathways via heterointerface. These architectural merits collectively endow the VS_2_@V_2_CT_X_ electrode with the structural resilience and conductivity needed for high‐rate and high‐areal‐capacity lithium storage. Notably, this synthesis route preserves the layered architecture of the underlying V_2_CT_X_, unlike conventional mixing routes VS_2_+V_2_CT_X_, which often disrupt structural integrity (Figure S5).

The density functional theory (DFT) calculations are a useful route to understand the in situ heterostructure property between metallic VS_2_ and V_2_CT_X_ MXene facilitates efficient ion/electron transport, which ultimately enhances the charge‐transfer kinetics and capacity of the heterostructure. This computational approach provides critical insights into the atomic‐level mechanisms driving the observed electrochemical performance. The computationally constructed heterostructure model (Figure 2e; Figure S6) exhibits a high density of electronic states at the Fermi level, confirming its metallic character according to total density of states (TDOS), which is primarily derived from the VS_2_ component (Figure 2f). This metallic nature is expected to significantly enhance electronic conductivity across the heterointerface. Four‐point probe measurements indicate that the VS_2_@V_2_CTx electrode exhibits superior electrical conductivity compared to pristine V_2_CTx (Figure 2g), which can be attributed to the in situ‐growth of VS_2_ on V_2_CTx, establishing efficient charge transport pathways and significantly enhancing charge migration within the electrode.

**FIGURE 3:**
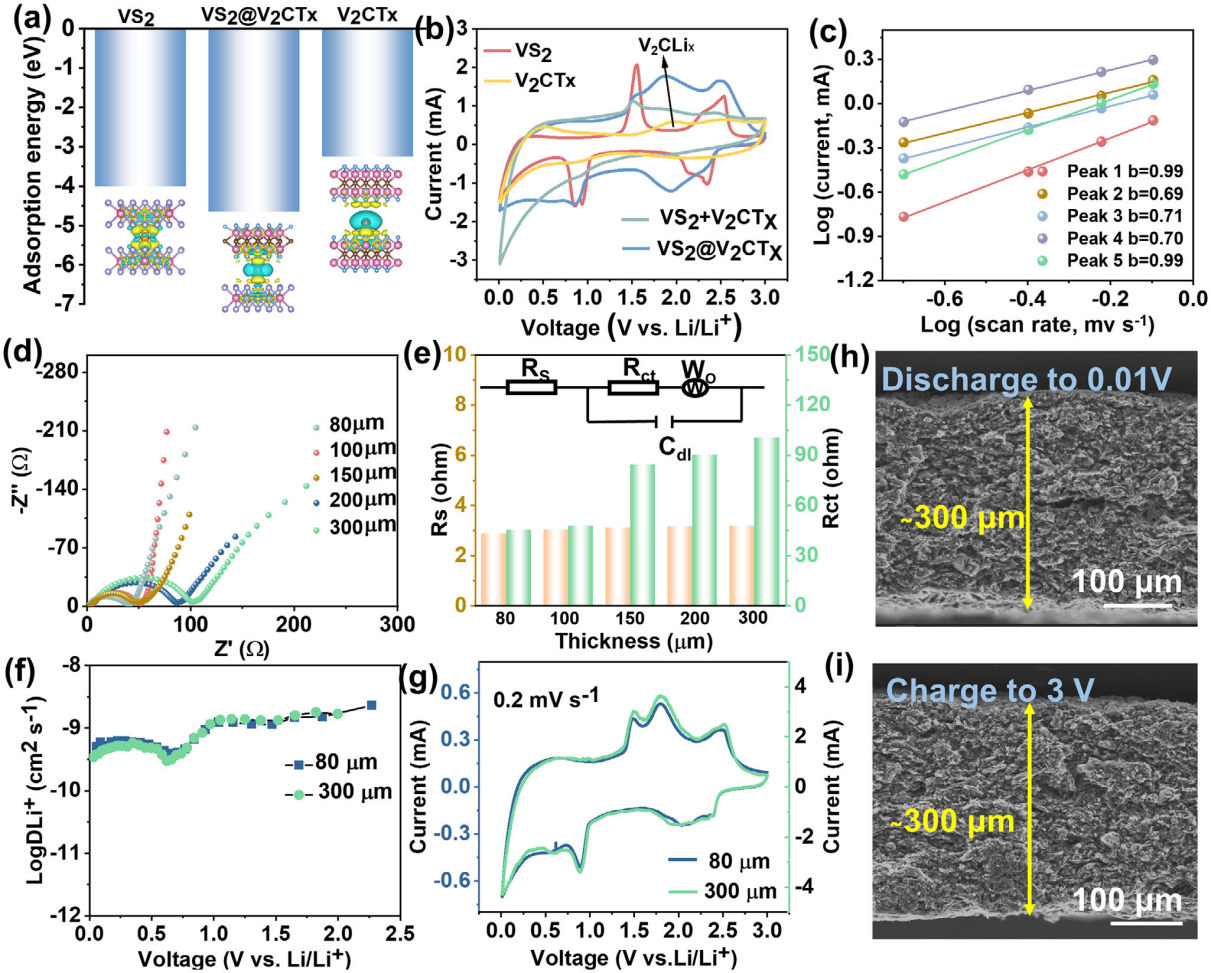
(a) Adsorption energy e of Li‐VS_2_, Li‐V_2_CTx and Li‐VS_2_@V_2_CTx heterostructure. (b) CV measurements feature the second cycle of VS_2_@V_2_CTx and VS_2_+V_2_CT_X_ under 0.4 mV s^−1^ in the voltage window between 0.01–3 V. (c) Capacitive effects are characterized by analyzing the CV curves from 0.2 to 0.8 mV s^−1^ based on i = av^b^, where the measured current i follows a power‐law relationship with the sweep rate v. (d) Nyquist plots of the EIS data for the VS_2_@V_2_CTx electrode with 80, 100, 150, 200 and 300 µm in Li half‐cell. (e) Equivalent circuit model and fitted values of the ohmic resistance (R_s_), SEI film resistance (R_SEI_), and charge‐transfer resistance (R_ct_) in Li half‐cell. (f) Calculated Li^+^ diffusion coefficients (DLi^+^) of the VS_2_@V_2_CTx electrode as a function of thickness, measured during the discharge state of the third cycle in a Li half‐cell at 25°C. (g) CV curves of VS_2_@V_2_CTx electrode with different thickness at 0.2 mV s^−1^. (h,i) Cross‐sectional SEM images of the VS_2_@V_2_CTx electrode after 10 cycles (1.3 mA cm^−2^), showing distinct charge/discharge states.

In addition, Li^+^ adsorption energy calculations (Figure 3a) reveal that the VS_2_@ V_2_CT_X_ heterostructure possesses a substantially lower adsorption energy (−4.67 eV) compared to pristine VS_2_ (−4.03 eV) and V_2_CT_X_ (−3.27 eV), indicating a thermodynamically favorable Li⁺ insertion process. Bader charge analysis further supports this observation, revealing simultaneous electron transfer across the heterointerface, establishing interpenetrating electron and ion transport pathways (Figure S7). This synergistic charge redistribution underpins the observed enhancement in electrochemical performance. The cyclic voltammetry (CV) profiles of VS_2_@V_2_CT_X_ exhibit a well‐defined redox pair centered near 1.9 V (vs. Li/Li^+^), which is absent in the physical mixture of VS_2_+V_2_CTx (Figure 3b). This distinct electrochemical feature suggests the formation of new redox‐active interfaces and significantly enhanced kinetics for both Li⁺ diffusion and electron transfer, contributing to improved capacity utilization. The charge storage mechanism by quantifying the capacitive and diffusion‐controlled contributions in both VS_2_@V_2_CT_X_ and VS_2_+V_2_CT_X_ electrodes. Using CV measurements at scan rates ranging from 0.2 to 0.8 mV s^−1^ and analyzing the relationship *i* = *k*_1_ *v* + *k*_2_ *v* ^1/2^, we observed that the b‐values for VS_2_@V_2_CT_X_ lie between 0.5 and 1.0, indicating a hybrid storage mechanism (Figures 3c; Figure S8). Notably, the pseudocapacitive contribution in VS_2_@V_2_CT_X_ reaches 84% at 0.8 mV s^−1^, substantially higher than that of the physical mixture (Figure S9), underscoring the dominance of fast surface‐controlled redox processes. This behavior is attributed to the rich population of electrochemically active surface sites, shortened charge transport distances, and efficient electron/ion interconnection enabled by the heterostructure. The Nyquist plots indicate reduced series and charge‐transfer resistances compared to the physical mixture VS_2_+V_2_CT_X_ (Figure S10), corroborating enhanced surface capacitance and more efficient Li⁺ diffusion [37]. Correspondingly, the Li⁺ diffusion coefficient (D_Li_), derived from Galvanostatic Intermittent Titration Technique (GITT) based on Fick's second law [38], 10^−9^ cm^2^ s^−1^ of VS_2_@V_2_CT_X_ is significantly higher for VS_2_+V_2_CT_X_ mixture (Figure S11), confirming the role of the VS_2_ nanosheets in forming fast ion‐diffusion channels. This architecture not only accelerates Li⁺ transport but also mitigates Li metal nucleation, supporting stable lithiation/delithiation and contributing to long‐term cycling stability [39, 40]. The electrochemical performance of VS_2_@V_2_CT_X_ in comparison to benchmark electrodes were evaluated, including bare V_2_CT_X_, pure VS_2_, and physically mixed VS_2_+V_2_CT_X_ with the thickness of 100 µm. In rate capability tests, the VS_2_@V_2_CT_X_ electrode exhibits reversible areal capacities of 3.1, 2.0, 1.5, and 0.8 mAh cm^−2^ at current density of 1.3, 2.6, 6.4, and 12.7 mA cm^−2^, respectively (Figure S12). Notably, when the current density is returned to 1.3 mA cm^−2^, the capacity rapidly recovers to 3.1 mAh cm^−2^, indicating minimal degradation and superior structural robustness. Long‐term cycling at 6.4 mA cm^−2^ (Figure S12c) shows that VS_2_@V_2_CT_X_ maintains >1.0 mAh cm^−2^ over 200 cycles with negligible fading, whereas control samples degrade significantly after 100 cycles. Collectively, these results confirm that the VS_2_@V_2_CT_X_ heterostructure—engineered via an in situ gas‐phase conversion strategy—realizes rapid electron/ion transport, high pseudocapacitive contribution, and robust interfacial contact.

**FIGURE 4:**
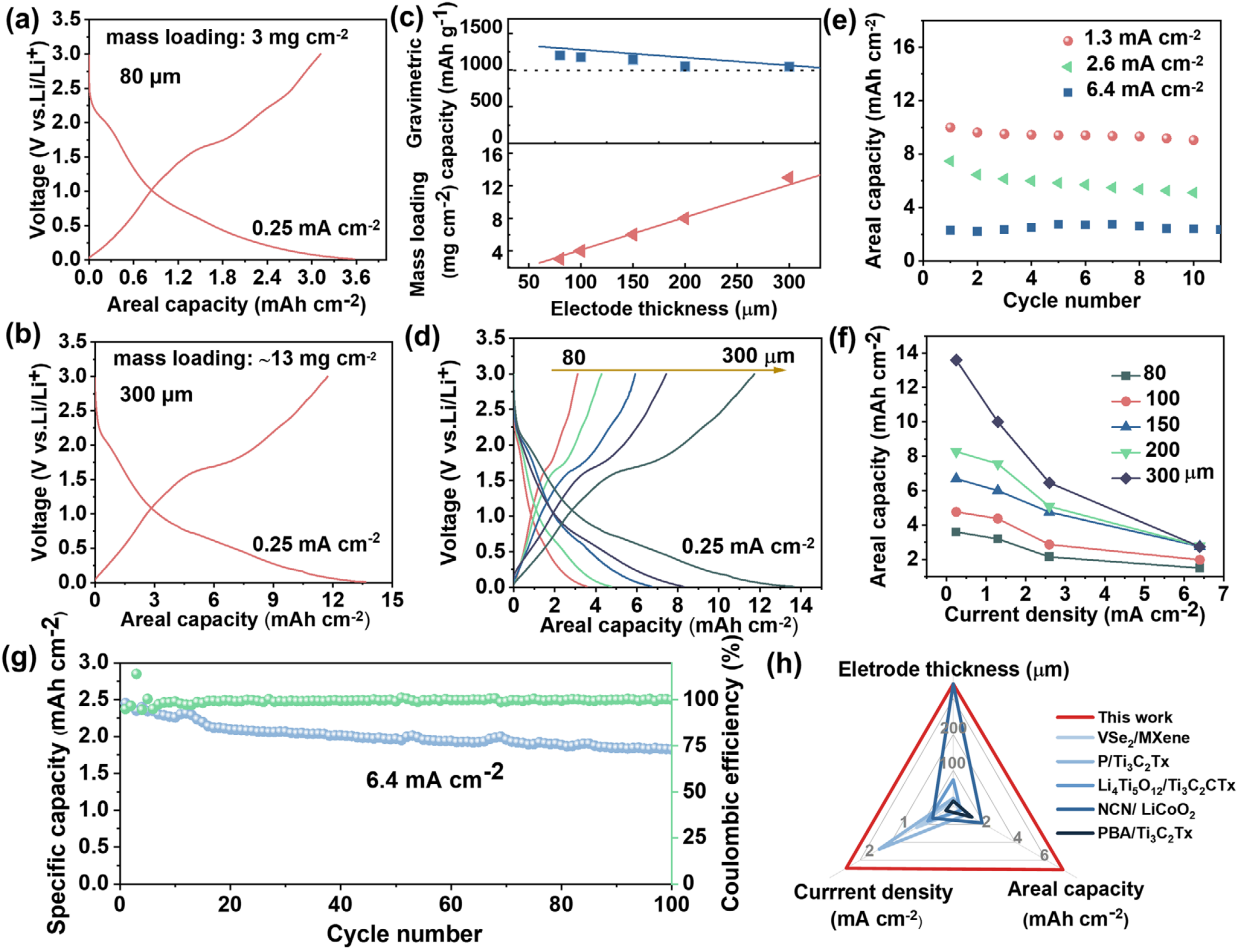
(a) and (b) are galvanostatic charge–discharge curves of VS_2_@V_2_CTx anodes with 80 and 300 µm and mass loading of ∼3 and 13 mg cm^−2^, respectively. (c) Gravimetric capacity and mass loading of VS@V_2_CTx electrode with different thickness. (d) Galvanostatic charge–discharge curves of VS_2_@V_2_CTx anodes with different thickness. (e) Rate performance for the VS_2_@V_2_CTx anodes under different current density with the thickness of 300 µm. (f) Dependence of areal capacity on electrode thickness and current density. (g) Long‐cycling performance of VS_2_@V_2_CTx electrode with the thickness of 300 µm at 6.4 mA cm^−2^. (h) Comparison of the recently reported battery performances of MXene or TMDC‐based cells.

We evaluated the electrochemical kinetics in the high‐loading electrode with different electrode thickness using electrochemical impedance spectroscopy (EIS), GITT and CV measurements. The EIS plot and fitting values of R_s_ and R_ct_ were quantified with an equivalent circuit presented in Figure 3d,e. As the electrode thickness increases from 80 to 300 µm, the resistance (R_s_, including electrolyte and electrode) remains consistently around 3 Ohm, while the charge‐transfer resistance (R_ct_ shows only a marginal increase from 45 to 100 Ohm, exhibiting a mere 122% increase in R_ct_ despite a 333% growth in electrode thickness. This indicates that the thicker electrode does not compromise overall battery resistance and is conducive to rapid charge‐transport dynamics. In addition, GITT analysis reveals that the ionic diffusion coefficients for both the 80 and 300 µm thick electrodes are comparable (Figure 3f), on the order of 10^−9^ cm^2^ s^−1^, suggesting that thick electrodes maintain rapid ion diffusion kinetics. In addition, the CV curves exhibit consistent redox peak positions across electrodes of different thicknesses (Figure 4g), suggesting that despite the increased thickness, efficient ion transport is maintained, thereby ensuring sufficient accessibility between ions and VS_2_@V_2_CTx and enabling capacity delivery. Cross‐sectional SEM images (Figure 3h,i) reveal that VS_2_@V_2_CTx electrode effectively accommodate large volume changes while maintaining a consistent electrode thickness.

The outstanding electrochemical performance is demonstrated to arise from heterostructure effects between the redox‐active V_2_CT_X_ matrix and the conductive VS_2_ domains, which collectively enhance charge transfer and ion transport kinetics. To unravel the origin of the exceptional rate capability and cycling stability observed in the VS_2_@V_2_CT_X_ heterostructure anode, we investigated the lithium‐ion kinetic behavior using ex situ EIS combined with distribution of relaxation times (DRT) analysis. Notably, the Nyquist plots (Figure S13a) demonstrate consistent impedance spectra upon charging/discharging, underscoring the excellent electrochemical reversibility of the VS_2_@V_2_CT_X_ electrode. The whole EIS spectra were converted as a relaxation‐based function γ(τ). The peaks at special relaxation time represent the related specialized electrochemical process, and the peak areas represent the impedance value [41, 42, 43]. Three dominant peaks emerge in the DRT spectra at relaxation times of 10^−5^–10^−4^ s, 10^−3^–1 s, and ∼10 s, corresponding to distinct electrochemical processes for Li‐VS_2_@V_2_CT_X_ (Figure S13b). The peaks at 10^−5^ to 10^−4^ s represent an irreversible evolution, which can be attributed to the solid electrolyte interphase (SEI) formation. The gradient value can be attributed to the ionic transport during the SEI formation process which is denoted as R_SEI_. There are three charge transfer processes at 10^−3^ to 1 s. Initially, the new charge transfer impedance (R_ct_1) located at of 10^−3^ s gradually decreases, which is corresponding to the delithiation process in Li_2_S to VS_2_. The second charge transfer processes (R_ct_2) located at of 0.01 s concurrence decrease, which indicates the transition from Li_2_S to S. The third charge transfer processes (R_ct_3) located at τ of 0.1 s rapidly emerge and decrease at the end of second plateau delithiation, which should indicate the transition from LiV_2_CT_X_ to V_2_CT_X_. These processes occur over increasingly longer time constants, consistent with progressive charge transfer dynamics. During the charging process (0.01 to 3 V), R_ct_ values rapidly decrease, indicating improved electronic conductivity and enhanced Li⁺ diffusion kinetics. The mirrored behavior during discharging (Figure S13c) further confirms the high reversibility of interfacial reactions in the VS_2_@V_2_CT_X_ heterostructure. Remarkably, the lowest R_ct_ is observed at the delithiated state, suggesting that the pristine VS_2_@V_2_CT_X_ interface is intrinsically conducive to lithium intercalation and charge transfer.

While DRT analysis reveals time‐resolved charge‐transfer processes, it lacks direct insight into specific redox events. Therefore, cyclic voltammetry (CV) was employed to elucidate the stepwise redox reactions and validate the participation of both intercalation and conversion mechanisms. As shown in Figure S14, the first and second CV curves recorded at 0.2 mV s^−1^ exhibit well‐defined, highly reversible redox couples (peaks 1–3). Minor shifts in peak position after the first cycle reflect initial electrochemical activation and the establishment of a stable SEI [44]. Highly reversible redox reaction couples emerge and contribute most of the capacity. The electrochemical behavior of the VS_2_ component follows a redox pathway involving Li^+^ intercalation and V^4+^ reduction, the canonical four‐electron reaction of pristine VS_2_ occurring near 0.8 V vs. Li/Li⁺.4*Li* ^+^ + *VS*_2_ + 4*e* ^−^ → *V* + *Li*_2_ *S*. The evolution of the valence state of sulfur provides direct evidence of its involvement in the redox reactions during cycling (Figure S15). This behavior is enabled by the close spatial proximity of vanadium atoms and Li_2_S species, which maintain intimate contact within the heterointerface and facilitate rapid charge exchange. The incorporation of V_2_CT_X_ MXene further modulates the redox behavior by stabilizing intermediate phases and enabling reversible participation of Li_2_S in the electrochemical process. This synergy gives rise to an areal‐capacity reaching 13.6 mAh cm^−2^, reflecting efficient utilization of both intercalation and conversion mechanisms. To substantiate the proposed redox mechanism, we conducted ex situ X‐ray diffraction (XRD) and transmission electron microscopy (TEM) analyses during the first electrochemical cycle at 100 mA g^−1^. XRD patterns (Figures S16 and S17) reveal the gradual attenuation and eventual disappearance of VS_2_ diffraction peaks during lithiation, followed by the appearance of Li_2_S signals upon delithiation—indicating a reversible conversion reaction. Crucially, the V_2_CT_X_ phase remains structurally intact throughout cycling, serving as a stable backbone. TEM observations after the first discharge to 0.01 V and subsequent recharge to 3.0 V (Figure S17a,b) confirm the formation of Li_2_S. In charge and discharge process, the persistence of V_2_CT_X_ underscores its role as a kinetically favorable, chemically stable support phase.

We paired the designed VS_2_@V_2_CTx electrodes (with the thickness of 80, 100, 150, 200 and 300 µm) with lithium metal anodes to assemble high‐performance batteries. The corresponding mass loadings were 3, 4, 6, 8 and 13 mg cm^−2^, respectively (Figure S18). Pure MWCNT‐based electrodes were also tested to isolate the contribution of the current collector (Figures S19 and S20). The MWCNT electrode displays initial discharge plateaus at 1.0 and 0.5 V, consistent with SEI layer establishment. Subsequent cycle elimination of these features demonstrates MWCNTs act exclusively as conductive matrices rather than active materials. The VS_2_@V_2_CTx electrode delivers a high discharge capacity of 1200 mAh g^−1^ with an areal capacity of 3.6 mAh cm^−2^ in low thickness (80 µm thickness, 3 mg cm^−2^; Figure 4a; Figure S17a). Remarkably, when scaled to industrial‐relevant thickness (300 µm, 13 mg cm^−2^), it maintains 1046 mAh g^−1^ gravimetric capacity while achieving a record areal capacity of 13.6 mAh cm^−2^ (Figure 4b), representing only 12.8% gravimetric capacity decay despite 333% increased electrode thickness (Figure 4c). Critically, the galvanostatic charge‐discharge profiles remain closely aligned for 80 µm (3 mg cm^−2^) and 300 µm (13 mg cm^−2^) electrodes, demonstrating near‐complete active material utilization and unimpeded reaction kinetics—direct evidence of the architecture's effectiveness in overcoming thick‐electrode transport limitations. To comprehensively assess performance across operational extremes, VS_2_@V_2_CTx electrodes with different thicknesses (from 80 to 300 µm) were cycled at current densities of 1.3–6.4 mA cm^−2^. The 300 µm electrode delivers 10.1, 8, and 2.3 mAh cm^−2^ at 1.3, 2.6, and 6.4 mA cm^−2^ respectively (Figure 4e), surpassing the 80 µm electrode (3.2 and 2.1 mAh cm^−2^ at 1.3/2.6 mA cm^−2^) by >215% in areal capacity (Supplementary Figure S18a). As expected, a gradual capacity fade is observed at high charge/discharge rates due to the substantial electrode thickness (Figure 4d,f). Nevertheless, a viable combination of capacity and rate performance is still attainable, which can be attributed to the highly conductive network formed by the segregated MWCNTs and VS_2_@V_2_CTx heterostructure that enables rapid charge distribution throughout the electrode. Critically, at the ultrahigh current of 6.4 mA cm^−2^, the thick electrode with 300 µm retains >1.8 mAh cm^−2^ after 100 cycles (Figure 4g). The as‐synthesized VS_2_@V_2_CT_X_ exhibits high‐rate performance, surpassing all control samples and other reported MXene/TMDCs composites or silicon‐based anodes. (Figure 4h; Table S2), demonstrating the viability of our heterostructure design for high‐power energy storage. Therefore, the VS_2_@V_2_CT_X_ heterostructures effectively decoupling areal capacity from current density constraints.

**FIGURE 5:**
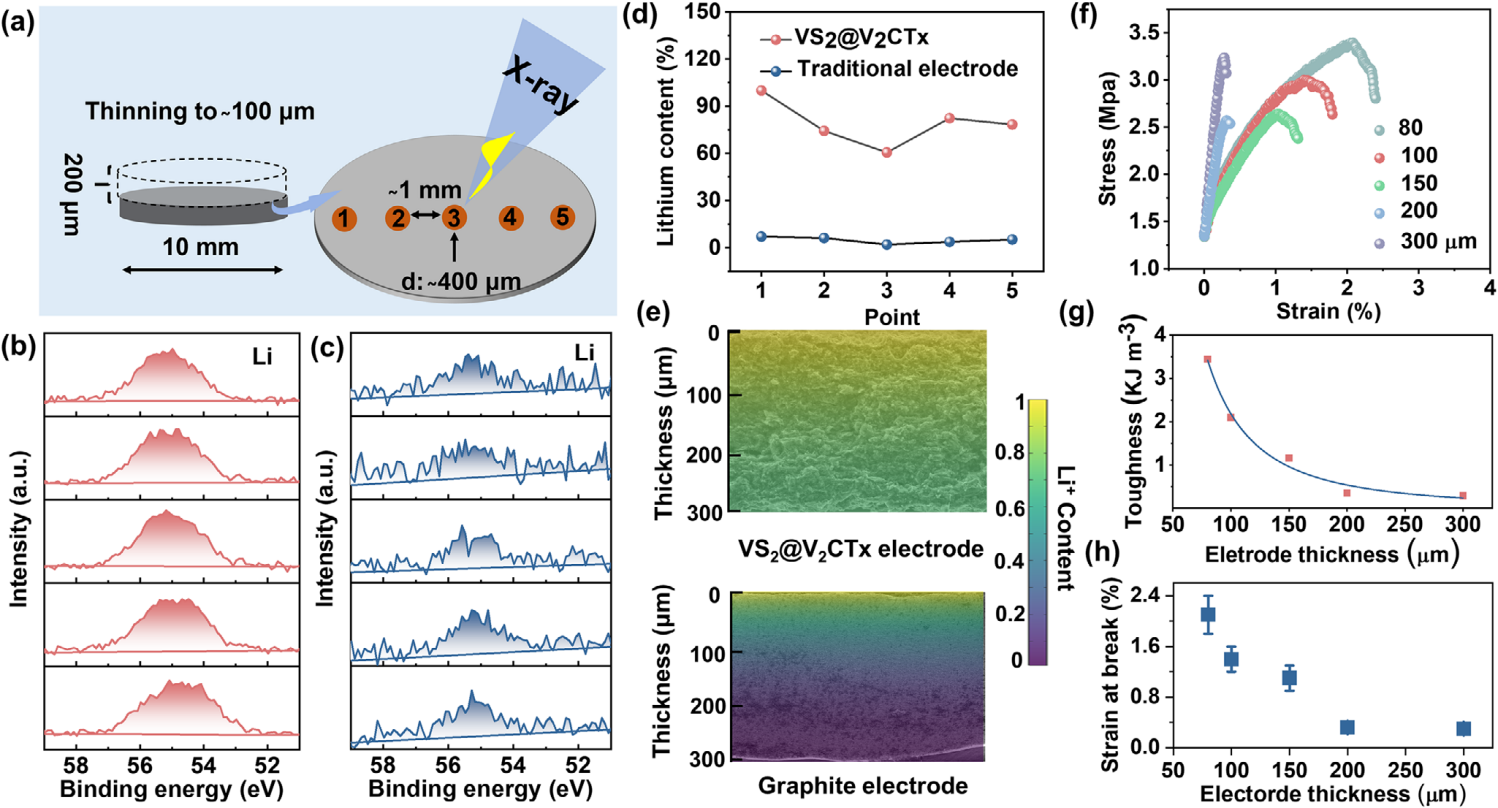
(a) Lithium‐ion concentration across thick electrodes via XPS analysis. (b) and (c) are Li XPS spectra at a depth of 200 µm within the VS_2_@V_2_CTx and graphite electrode, respectively. (d) Li^+^ relative content of VS_2_@V_2_CTx and traditional electrode. (e) Schematic illustrating the distinct Li^+^ diffusion kinetics simulation in the VS_2_@V_2_CTx versus the slurry‐cast graphite electrode, based on XPS depth‐profiling results. (f) Representative stress–strain curves for VS_2_@V_2_CT_X_ electrodes with different thickness. (g) Tensile toughness versus electrode thickness. (h) Strain at break for composite films versus electrode thickness.

The VS_2_@V_2_CTx heterostructure effectively resolves the thick electrode paradox by constructing a 3D highway with interpenetrating, low‐tortuosity pathways for simultaneous and rapid transport of electrons and Li^+^ ions. To quantitatively validate this core advantage, we systematically compared a 300 µm‐thick VS_2_@V_2_CTx electrode against a conventional graphite electrode (fabricated on copper current collectors) after lithiation (Figure S21). Lithium content was measured at approximately 1 mm intervals along the electrode diameter after thinning to 100 µm, (Figure 5a; Figure S22). The VS_2_@V_2_CTx electrode exhibited a uniform and pervasive distribution of Li^+^ across all analyzed points (Figure 5b). In stark contrast, the conventional graphite electrode showed a sharp decline in Li^+^ signal, indicating severely limited Li^+^ penetration (Figure 5c). The superior lithium‐ion transport kinetics within the VS_2_@V_2_CTx heterostructure are quantitatively confirmed by depth‐profiling analysis (Figure 5d; Table S1). Most strikingly, at a critical depth of 200 µm—where conventional graphite electrodes suffer from severe ionic depletion—the VS_2_@V_2_CTx architecture maintains a Li^+^ concentration 12‐fold higher (Figure 5e). This stark contrast underscores the role of its low‐tortuosity, interpenetrating ion channels in enabling uniform reaction activity and overcoming the fundamental limitations of thick electrodes.

To fully realize the electrochemical potential of the VS_2_@V_2_CT_X_ heterostructure as a high‐performance battery electrode, further enhancement of its mechanical properties and structural integrity is essential. We contend that the incorporation of MWCNTs offers distinct advantages for mechanical reinforcement, owing to their capability to form interconnected, entangled networks with the accordion‐like architecture of V_2_CT_X_ MXene. Representative stress–strain curves are shown in Figures 5h–j, when the electrode thickness is 80 µm, the strain reaches 2.1%. As the thickness increases to 300 µm, the electrode withstands stress up to 3.25 MPa before fracture, indicating the presence of superior cohesion and synergistic interaction between MWCNTs and VS_2_@V_2_CT_X_ heterostructure. Of particular interest is the tensile toughness, a property strongly correlated with electrode mechanical stability, as demonstrated in previous studies where tough electrodes exhibited high crack resistance [45]. Remarkably, even at an electrode thickness of 300 µm, the material retains significant toughness, underscoring its structural resilience under high‐stress conditions (Figure 5i). Importantly, unlike traditional electrodes, the integrated MWCNTs and VS_2_@V_2_CTx electrode exhibits a correlated relationship between toughness and electrical conductivity, resulting in a simultaneous enhancement of both mechanical resilience and charge transport properties compared to conventional systems.

The answer for how the electrode interface evolves during cycling could help reveal the mechanisms responsible for the exceptional cycling stability at high areal capacities, which provides insight into the factors that preserve the integrity and functionality of the anode over extended use. EIS measurements of VS_2_@V_2_CT_X_ anodes at different cycling stages and corresponding DRT analysis reveal minimal change in SEI resistance (R_SEI_) (Figure S23a–f), suggesting excellent chemical and electrochemical stability of the interfacial layer over prolonged operation. This stable SEI minimizes active material loss and maintains structural cohesion, which is critical for long‐term cycling. Cross‐sectional SEM images of the VS_2_@V_2_CT_X_ electrode after 100 cycles reveal a uniformly conformal SEI layer throughout the entire 300 µm thickness (Figure 5e,f), providing further evidence of its complete and homogeneous lithiation. In addition, and XPS (Figure 5d) further confirm the presence of LiF and Li_2_CO_3_ as the dominant inorganic SEI components on cycled VS_2_@V_2_CT_X_ electrode. These species originate from the decomposition of LiPF_6_ and electrolyte solvents, and synergistically stabilize the electrode/electrolyte interface [46, 47]. Specifically, LiF imparts high ionic conductivity and acts as a barrier to electron tunneling, while Li_2_CO_3_ contributes to passivating the surface and reducing parasitic reactions [48]. The LiF‐rich SEI also lowers the Li^+^ diffusion barrier and Li^+^ adsorption energy, thus facilitating rapid charge transport and suppressing dendritic growth. Moreover, the high electrochemical stability and wide bandgap of LiF enable uniform Li plating/stripping, essential for maintaining high Coulombic efficiency at elevated current densities [49]. Altogether, the robust SEI enriched in LiF and Li_2_CO_3_ serves as a critical component in enabling high‐rate operation and long cycle life [50]. It complements the conductive and ion‐permeable framework of the VS_2_@V_2_CT_X_ heterostructure, collectively ensuring dendrite‐free cycling, stable electrode morphology, and high‐areal‐capacity retention under demanding electrochemical conditions.

To evaluate the real‐world applicability of VS_2_@V_2_CT_X_ anode, full cells were assembled with LiFePO_4_ and LiNi_0.8_Co_0.1_Mn_0.1_O_2_ (NCM811) cathodes. The electrochemical characterization of VS_2_@V_2_CT_X_||LiFePO_4_ full cell is shown in Figure S24. The anode is made of direct VS_2_@V_2_CT_X_ on the MWCNTs_._ As seen from Figure S24a, the VS_2_@V_2_CT_X_||LiFePO_4_ full cell as in the normal LIB cells above present the discharge capacities of 150, 124, 101 and 50 mAh g^−1^ at 0.5C, 1C, 2C and 4C, respectively. When the current density returns to 0.2C from 10C, ∼100% of the initial capacity is recovered. Upon returning to 0.2C, nearly 100% of the initial capacity is recovered, highlighting the fast kinetics and structural stability of the anode. Long‐term cycling at 0.2C yields a capacity retention of 92% after 100 cycles with near‐unity Coulombic efficiency. The corresponding Coulombic efficiency gradually increases to 100% after a few cycles and then becomes stable. At a high rate, the VS_2_@V_2_CT_X_||LiFePO_4_ full cell also present the excellent cycling performance. As shown in Figure S24c,d, the VS_2_@V_2_CT_X_||LiFePO_4_ full cell exhibit more stable long‐term cycling performance, it delivers the retained capacity of 90 mAh g^−1^ and the capacity retention of 81% after 600 cycles. Furthermore, the cyclic performance of the VS_2_@V_2_CT_X_||NCM811 full cell was tested at 1C and 2C (1C = 200 mAh g^−1^), respectively (Figure S25). the VS_2_@V_2_CT_X_|| NCM811 full cell shows an average discharge plateau of ≈3.2 V. Even prolonging the battery cycles up to 100 cycles, a capacity retention is about 100% and 75% was achieved in VS_2_@V_2_CT_X_||NCM811 full cell under the current density of 1C and 2C, respectively. These results unequivocally establish the potential of VS_2_@V_2_CT_X_ as a practical high‐capacity anode for lithium‐ion batteries.

## 3 Conclusion

In summary, we report an in situ gas‐phase conversion strategy for constructing VS_2_@V_2_CT_X_ heterostructures as high‐performance anode for lithium‐ion batteries. By integrating these heterostructures with MWCNTs based current collectors, we achieve exceptional areal capacities and long‐term cycling stability, marking a significant advance in the design of scalable, high‐areal‐capacity electrode. Notably, areal capacities reach capacity of 13.6 mAh cm^−2^ with the thickness of 300 µm, with a mass loading of 13 mg cm^−2^, representing only 12.8% gravimetric capacity decay despite 333% increased electrode thickness, and exceed 1.8 mAh cm^−2^ after 100 cycles under high current densities of 6.4 mA cm^−2^. In full‐cell assemblies with LiFePO_4_ cathodes, the VS_2_@V_2_CT_X_ anodes demonstrate exceptional stability, retaining 81% of their initial capacity after 600 cycles at 2C. The exceptional areal capacity and cycling stability stem from the VS_2_@V_2_CT_X_ heterostructures via in situ gas‐phase conversion, which enhance conductivity (2304 S m^−1^), establish interpenetrating electron/ion transport pathways, accelerate Li⁺ diffusion (D_Li_^+^≈10^−9^ cm^2^ s^−1^), and leverage interfacial pseudocapacitance (84% capacitive contribution at 0.8 mV s^−1^). Furthermore, full lithiation in ultra‐thick electrodes, the improved mechanical toughness, ion‐permeable LiF/Li_2_CO_3_ dual‐phase SEI—characterized by enables ultrafast charge transfer, critical for sustaining cycling stability at high current density. Complementary DFT calculations support the experimental findings, demonstrating reduced Li^+^ adsorption and charge redistribution across the heterointerface. The combined advantages of structural design, kinetic enhancement, and mechanical stability highlight the VS_2_@V_2_CT_X_ system as a paradigm for smart materials design, effectively breaks the critical paradox between thick electrodes and charge transport efficiency. These insights lay a robust foundation for the development of high‐energy‐density lithium‐ion batteries poised for large‐scale applications.

## Conflicts of Interest

The authors declare no conflicts of interest.

## Supporting information




**Supporting file**: advs73475‐sup‐0001‐SuppMat.docx.

## Data Availability

The data that support the findings of this study are available from the corresponding author upon reasonable request.
