# Supplementary material for: Breaking the Thick Electrode Paradox With an in situ VS2@V2CTx MXene Heterostructure for High‐Areal‐Capacity Batteries
Source: Adv Sci (Weinh). 2025 Dec 21;13(10):e22300. doi: 10.1002/advs.202522300 (PMC12915098; doi:10.1002/advs.202522300)
Supplement: Supplementary file 1 — Supporting file: advs73475‐sup‐0001‐SuppMat.docx. [file ADVS-13-e22300-s001.docx]

**Breaking the Thick Electrode Paradox with an In-situ VS_2_@V_2_CTx MXene Heterostructure for High-Areal-Capacity Batteries**

*Lirong Wang*, *Jiulong Li, Ye Chen*, *Cheng Zhang*, *Youquan Jiang*, *Chaoyu Chen*, *Haonan Song*, *Peigen Zhang**, *Sheng Liu**, *Zhaodong Li**

L. Wang, J. Li, Y. Jiang, C. Chen, H. Song, S. Liu, Z. Li

School of Integrated Circuits, Wuhan University, Wuhan, 430072, China

E-mail: [shengliu@whu.edu.cn](mailto:shengliu@whu.edu.cn), [Zhaodong_Li@whu.edu.cn](mailto:Zhaodong_Li@whu.edu.cn)

Y. Chen, C. Zhang, P. Zhang

School of Materials Science and Engineering, Southeast University, Nanjing, 211189, China

E-mail: [zhpeigen@seu.edu.cn](mailto:zhpeigen@seu.edu.cn)

**Methods**

**Preparation of V_2_CT_X_ MXene**

Add 1 g LiF and 20 ml 6M concentrated hydrochloric acid into 100 ml polytetrafluoroethylene (PTFE) bottle, dissolve slowly under continuous magnetic stirring at 35 ℃, and add 1 g V_2_AlC powder. Then, the sample was heated in an oil bath at 90 ℃ for 72 h to obtain a black precipitate. Then, the black precipitate is repeatedly centrifuged and washed with deionized water until the pH is about 6~7, and the black precipitate solid is collected as V_2_CT_X_. The prepared V_2_CT_X_ MXene was freeze-dried overnight at -40 ℃. V_2_CT_X_ powders then mixed with 0.025 g multi-wall carbon nanotubes (MWCNTs) in ethanol solution to mix evenly by ultrasonic suction. MWCNTs with high conductivity acts as a current collector in the prepared electrode. Then filter V_2_CT_X_ and MWCNTs mixture into membrane. Then, the membrane was cut into a disc with a diameter of 10 mm as negative electrode of Li-ion battery.

**VS_2_@V_2_CT_X_ heterostructure built by in-situ gas phase conversion**

8 g thioacetamide (TAA) is placed upstream of the quartz tube, and a disc loaded with V_2_CT_X_ is placed 20 cm downstream. The temperature rises to 700 ℃ for 1 min, 2 h and 4 h with the heating rate of 4 degrees per minute, then cool naturally to room temperature.

**Hydrothermal synthesis of VS_2_**

Thioacetamide (C_2_H_5_NS, 64 mmol) and 8 mmol Ammonium Metavanadate (H_4_NO_3_V) were dissolved in a solution containing 50 mL of deionized water and 12 mL of Ammonia (NH_3_·H_2_O). Stir for 30 minutes, then transfer to a 100 mL PTFE reactor. After 24 hours of treatment in a hot air oven at 180 ℃ and natural cooling, the obtained black product was washed repeatedly with deionized water and dried in an 80 ℃ oven for 12 hours. As mentioned above, VS_2_ and MWCNTs were mixed and filtered into a membrane to prepare the electrode.

**DFT Calculation**

The first-principles calculation was conducted in the density functional theory (DFT) framework in the Vienna Ab initio Simulation Package (VASP). V_2_CTx was simplified as V_2_CO_2_ because T_X_ terminations were complex and mainly composed of oxygen-containing functional groups^[1]^. The bilayer models of VS_2_, V_2_CO_2_, and VS_2_@V_2_CO_2_ were adopted as approximations of the studied materials in this work. The projector augmented wave (PAW) method together with the generalized gradient approximation constructed by Perdew–Burke–Ernzerhof (GGA-PBE) was applied for the exchange-correlation energy function. The cutoff energy was set to 500 eV in this work, and a Γ-point k-grid scheme was used due to the large size of the model. The convergence criterion for the electronic self-consistent iteration was set to 10^–4^ eV, and the atomic positions were fully relaxed until the force on each atom was <0.02 eV Å^–1^.

**Numerical modeling**

The diffusion of Li ions was taken as a time-dependent process and was governed by Fick’s second law. In COMSOL™, the module of transport of diluted species was utilized to model the diffusion process. The flux of Li ions was defined by XPS data obtained from the experiment (Supplementary Table 1). We define the comparison of Li+ concentration in the thickness direction between slurry-cast graphite electrode and VS_2_@V_2_CTx electrodes under complete lithiation.

**Characterizations**

A Tescan MIRA scanning electron microscopy (SEM) equipped was utilized to provide morphological views operating at 5 kV. Raman spectra were collected using a XploRA Plus Raman microscope. A 473-nm solid-state laser as the excitation source. The excitation light with a power of 2.5 mW was focused onto the sample by a 100X objective lens (N.A. = 0.9). HR-TEM imaging was conducted using a JEM-F200 Japan Titan Themis Z transmission electron microscope (TEM) equipped with super energy spectrometer (dual energy spectrum probe) jed-2300t Gatan OneView camera at 200 kV.

**Electrochemical characterization**

Anodes were directly mixed with MWCNTs current collector without additional processing steps. The loading level of spatially homogeneously VS_2_@V_2_CT_X_, was ∼3 mg cm^-2^,4 mg cm^-2^, 6 mg cm^-2^, 8 mg cm^-2^, 13 mg cm^-2^ (electrode area is 0.785 cm^-2^). CR2032 coin-type cells were assembled in n argon-filled glove box containing pure Li metal foil as the counter electrode. The electrolyte was 1.0 M Lithium hexafluorophosphate (LiPF6) in a mixture of ethylene carbonate (EC) and dimethyl carbonate (DMC) with a volume ratio of 1:1 (Kluthe). Polypropylene with a thickness of 25 µm was used as a separator. Galvanostatic cycling was conducted on a computer controlled Landt battery test system at different current densities (1.3, 2.6, 6.4 and 12.7 mA cm^-2^) in a potential range of 0.01−3.0 V vs. Li/Li^+^. The cyclic voltammetry (CV) tests were carried out to examine the electrode reaction under the scan rate of 0.2, 0.4, 0.6 and 0.8 mV s^−1^ with a potential range of 0.01−3.0 V vs. Li/Li^+^. Electrochemical impedance spectra (EIS) were recorded in a frequency range from 10^5^ to 0.01 Hz. At the same time, the disturbance amplitude was 5 mV. CV and EIS were conducted with a CHI600F electrochemical workstation. All electrochemical measurements were under constant 25 °C. The anode is binder-free and carbon-free.

**Lithium ion concentration testing in thick electrodes**

Thick electrodes (VS_2_@V_2_CT_X_ and traditional graphite electrode), initially prepared at a uniform thickness of 300 μm, were thinning to 100 μm for XPS analysis. Lithium content was measured at approximately 1 mm intervals along the electrode diameter.

**Mechanical measurements**

Mechanical measurements were conducted from free-standing samples using a Zwick Z0.5 Pro-Line Tensile Tester at a strain rate of 0.5 mm min^−1^. Each data point was obtained by averaging the results from three independent measurements.

**VS_2_@V_2_CT_X_****||LiFePO_4_ full cell measurement**

VS_2_@V_2_CT_X_||LiFePO_4_ full cell measurement VS_2_@ V_2_CT_X_ works as the anode in the full cell LIB configuration, while the commercial LFP (lithium iron phosphate, LiFePO_4_) and NCM811 (Lithium nickel cobalt manganate, LiNi_0.8_Co_0.1_Mn_0.1_O_2_) worked as the cathode in a 2032 coin-type cell. Electrolyte is 1 M LiPF_6_ in EC:DMC 1:1 solution. The cathodes consisted of 90 wt% LFP and NCM811, 5 wt% polyvinylidene fluoride (PVDF), and 5 wt% super-P on Al current collectors. The anode and cathode active materials loading was adjusted to ensure that VS_2_@V_2_CT_X_ anode capacity was 10% higher than the cathode. The active material loading was 6 mg cm^−2^ for the VS_2_@ V_2_CT_X_ anode, 4 mg cm^−2^ for the LFP and NCM811 cathode, respectively.


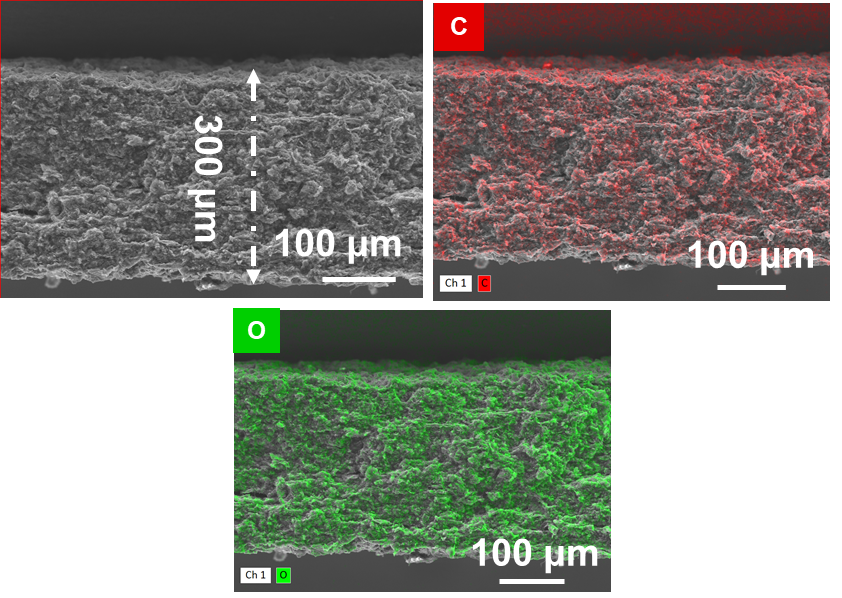


Supplementary Figure 1. Cross-sectional scanning electron microscopy (SEM) and EDS images of the integrated electrode of V_2_CT_X_ with MWCNts.


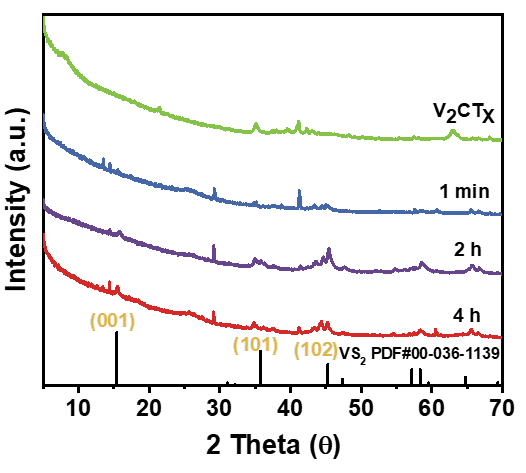


Supplementary Figure 2. Schematic illustrations of different holding time at 700 ℃ corresponding to the resultant XRD patterns.


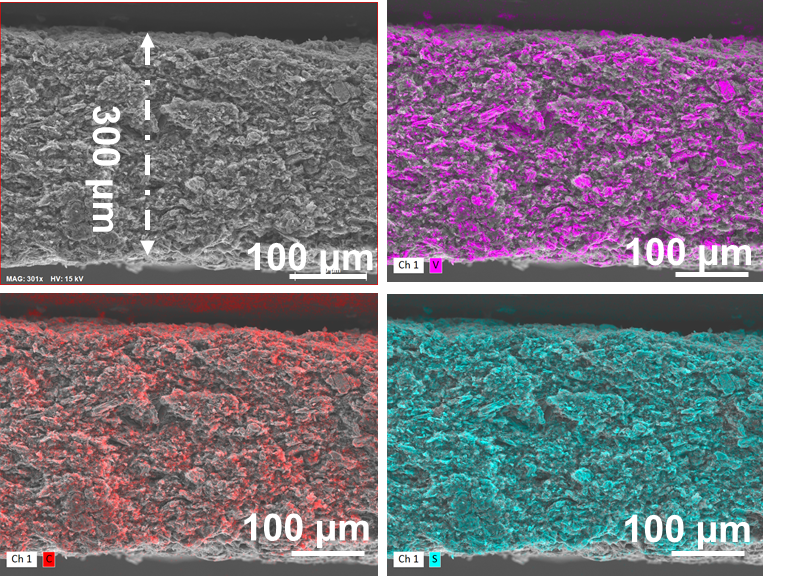


Supplementary Figure 3. Cross-sectional scanning electron microscopy (SEM) and EDS images of the integrated electrode of VS_2_@V_2_CT_X_.


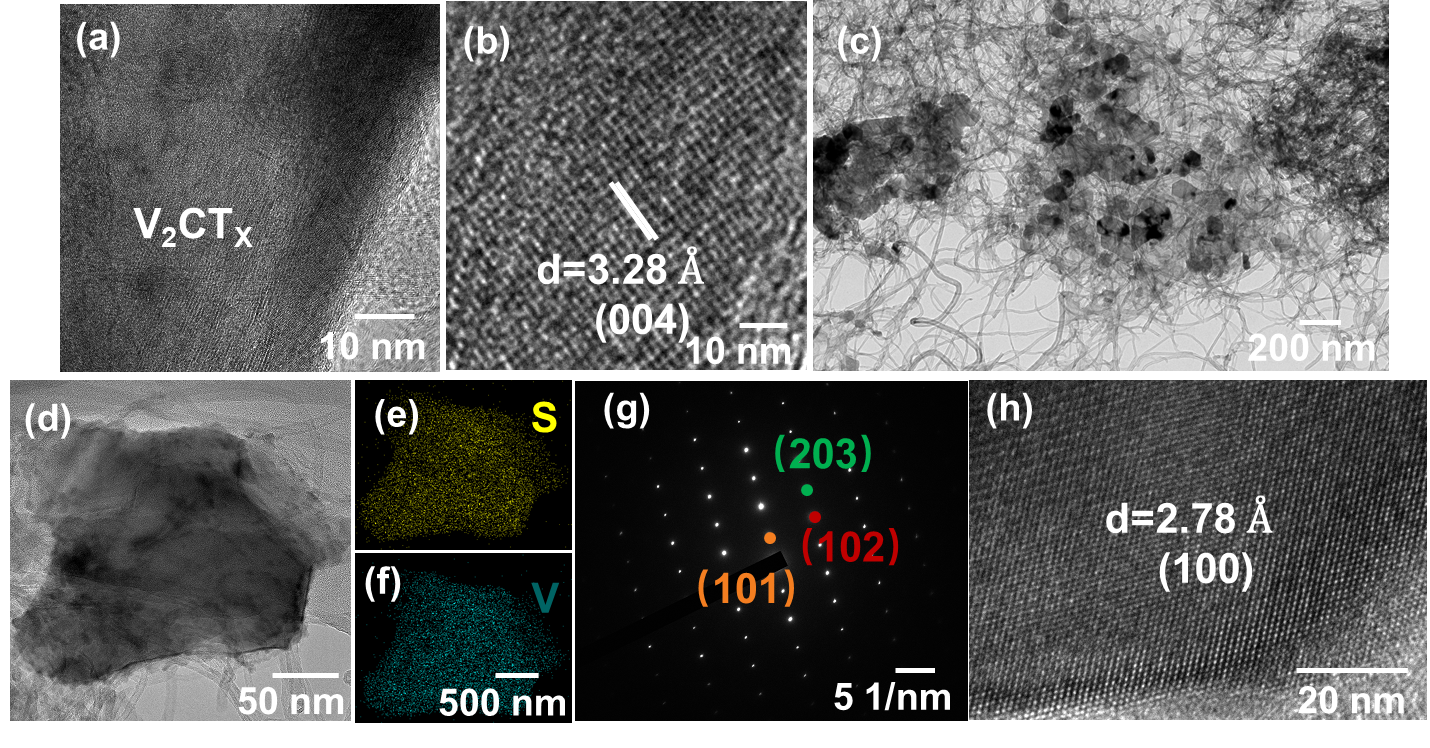


Supplementary Figure 4. (a) is TEM image of V_2_CT_X_ in VS_2_@V_2_CT_X_ and (b) is enlarged view of (a). (c-h) are TEM images of VS_2_ in VS_2_@V_2_CT_X_ after ultrasonic dispersion, (d) is TEM image of VS_2_ nanosheets, (h) is higher resolution TEM image showing the large crystalline domain area of VS_2_, (e) and (f) are EDS mapping of VS_2_, (g) is selected area electron diffraction pattern, the sharp diffraction spots indicate that VS_2_ has good crystallinity.


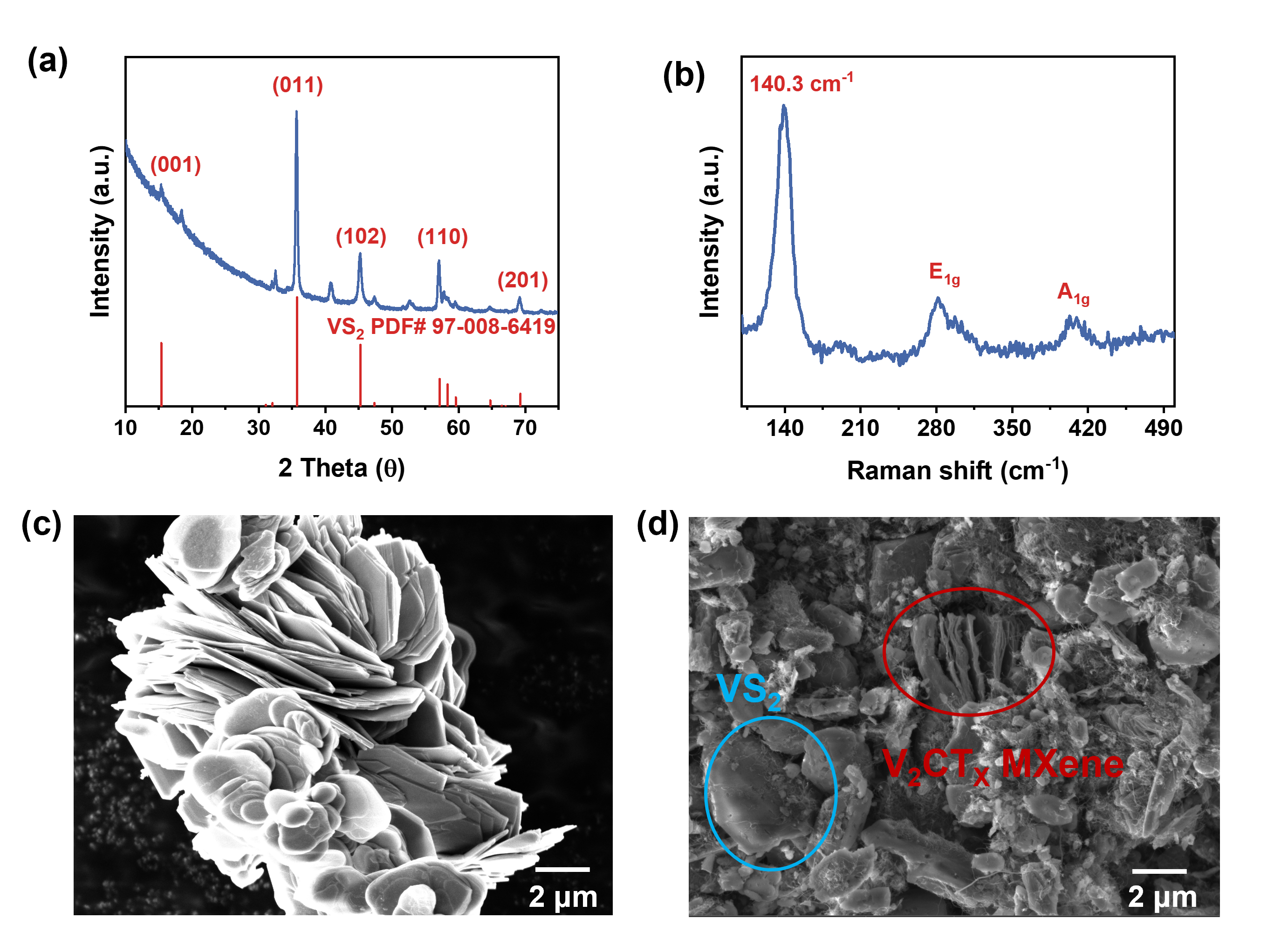


Supplementary Figure 5. VS_2_ synthesized by hydrothermal method. (a) XRD patterns of VS_2_ (the formation of VS_2_ is confirmed by appearance of the characteristic peak (001), (011) and (102) of VS_2_. (b) Raman spectrum of VS_2_ (as shown, the characteristic bands at 280 and 402 cm^−1^ should be assigned to the in-plane E_1g_ (the opposite vibration of the two S atoms with respect to the V atom) and out-of-plane A_1g_ (the symmetric displacement and compressive force of S atoms along the c-axis) vibration modes, respectively^[2]^). (c) SEM image of VS_2_. (d) electrode surface made of VS_2_, V_2_CT_X_ MXene and MWCNTs mixed by ultrasonic filtration (accordion shaped V_2_CT_X_ MXene mixed with VS_2_ sheets).


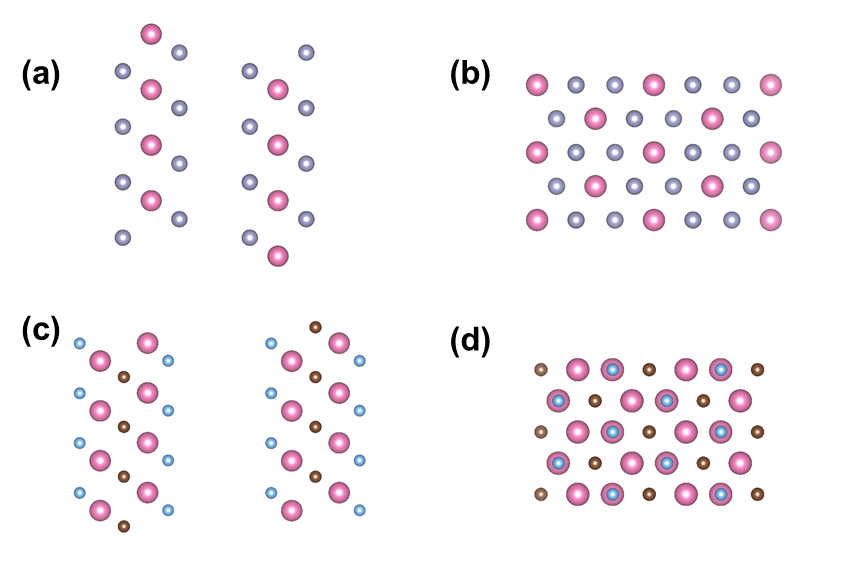


Supplementary Figure 6. Computational structural model of VS_2_ (a-b) and. V_2_CT_X_ (c-d).


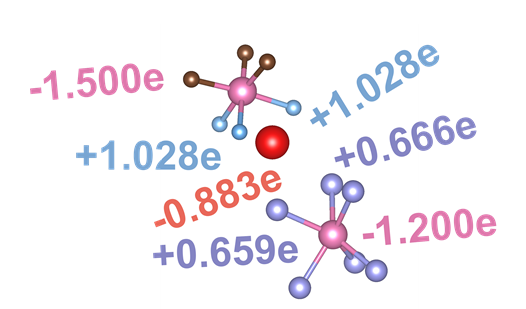


Supplementary Figure 7. Partial atomic gain and loss of electrons of VS_2_@V_2_CT_X_ by Bader charge analysis.


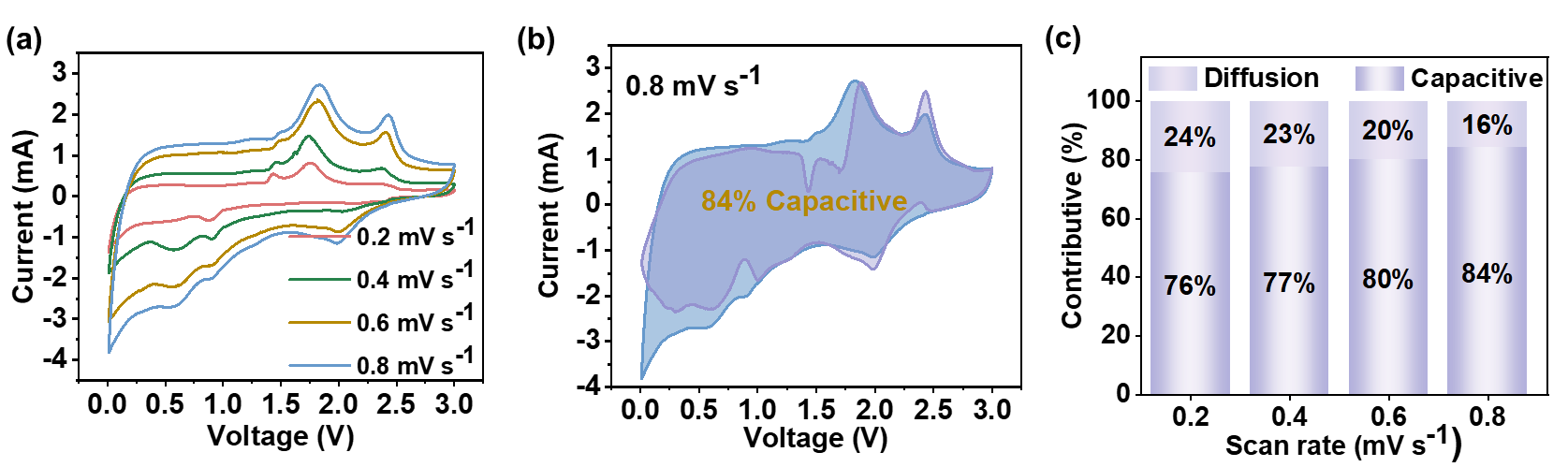


Supplementary Figure 8. (a) CV curves of VS_2_@V_2_CT_X_ electrode at different sweep rates. (b) Capacitive effects are characterized by analyzing the CV curves at various sweep rates based on i = av^b^, where the measured current i follows a power-law relationship with the sweep rate v. (f) The percentage of pseudocapacitive contribution at different 0,8 mV s^-1^.


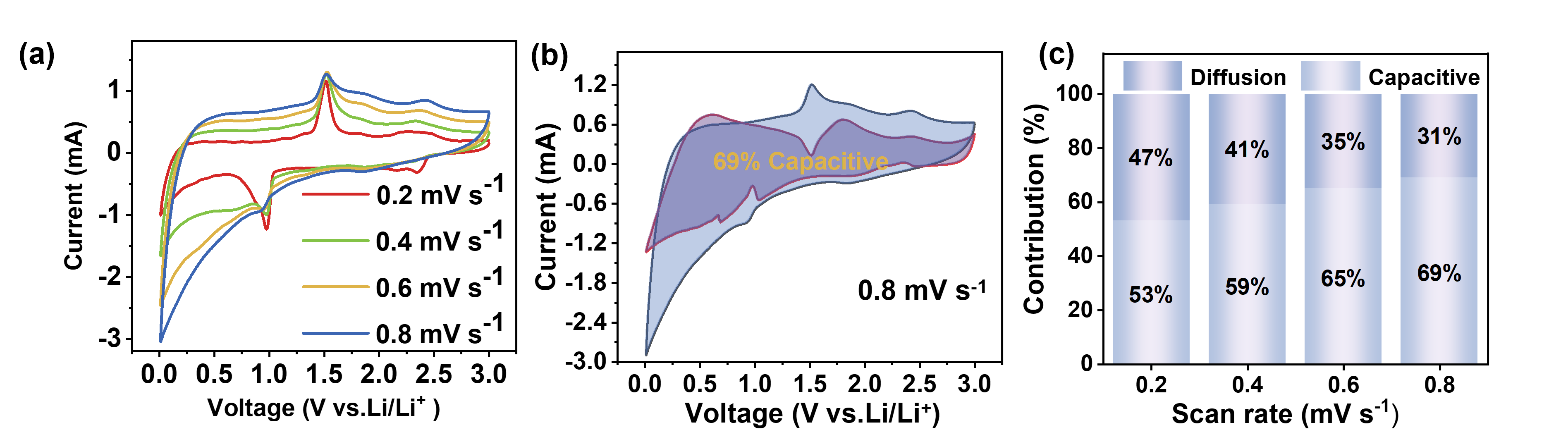


Supplementary Figure 9. Pseudocapacitive energy storage ratios to the total charge storage in reference samples. (a) CV curves of VS_2_+V_2_CT_X_ mixture at various sweep rate. (b) Capacitive and diffusion controlled charge storage contributions for architected VS_2_+V_2_CT_X_ cycled in a Li-ion electrolyte at a scan rate of 0.8 mV s^−1^. (c) The percentage of pseudocapacitive contribution at different scan rates.


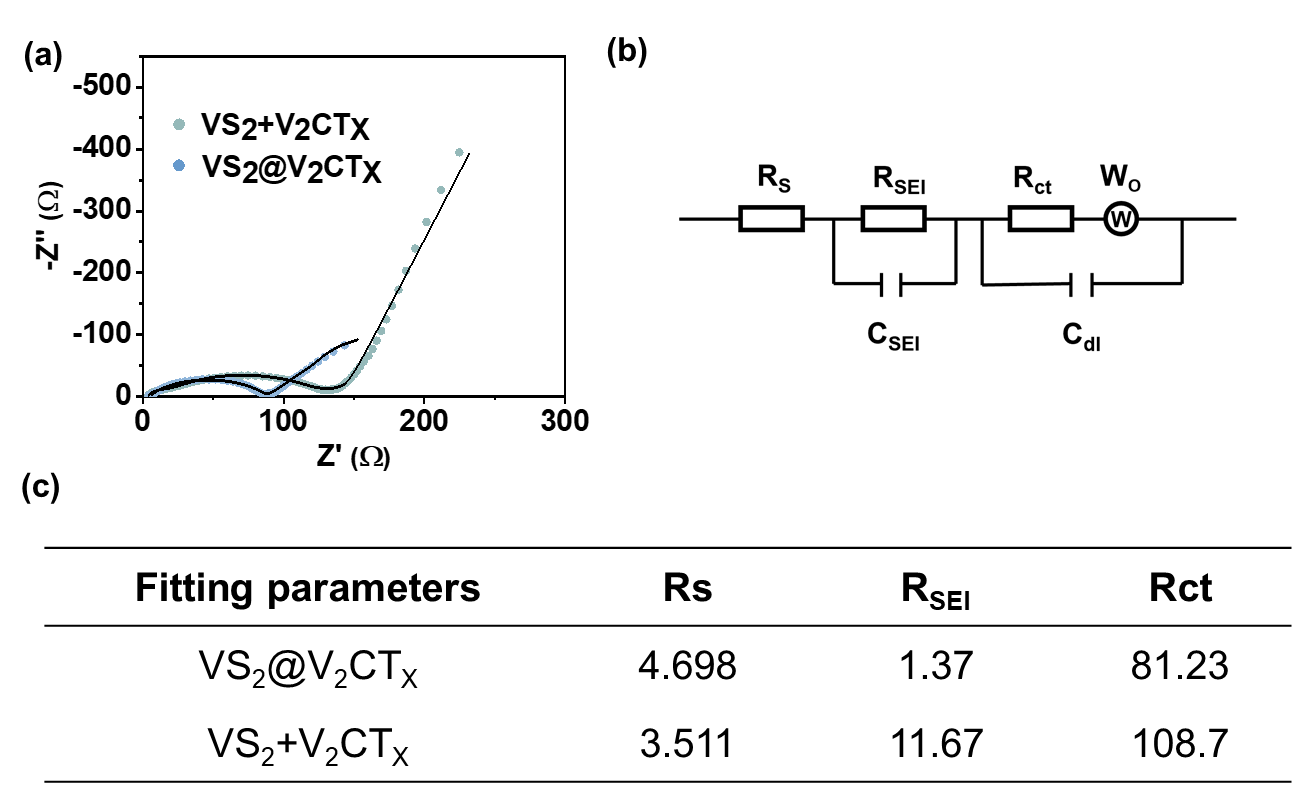


Supplementary Figure 10. (a) Nyquist plots of VS_2_@V_2_CT_X_, VS_2_+V_2_CT_X_ mixture electrode. (b) The equivalent circuit model of VS_2_@V_2_CT_X_ electrode. (c) Values of Re, RSEI and Rct are obtained by fitting data to (b).


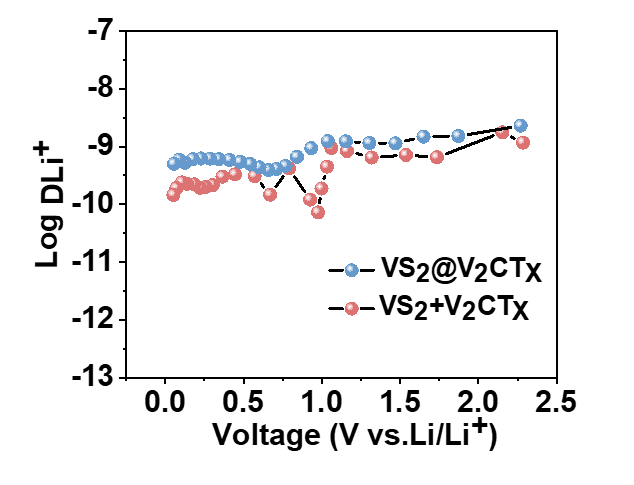


Supplementary Figure 11. Diffusion coefficient values (D_Li_^+^) in terms of logarithms to the base 10 of VS_2_@V_2_CT_X_ and VS_2_+V_2_CT_X_ mixture.


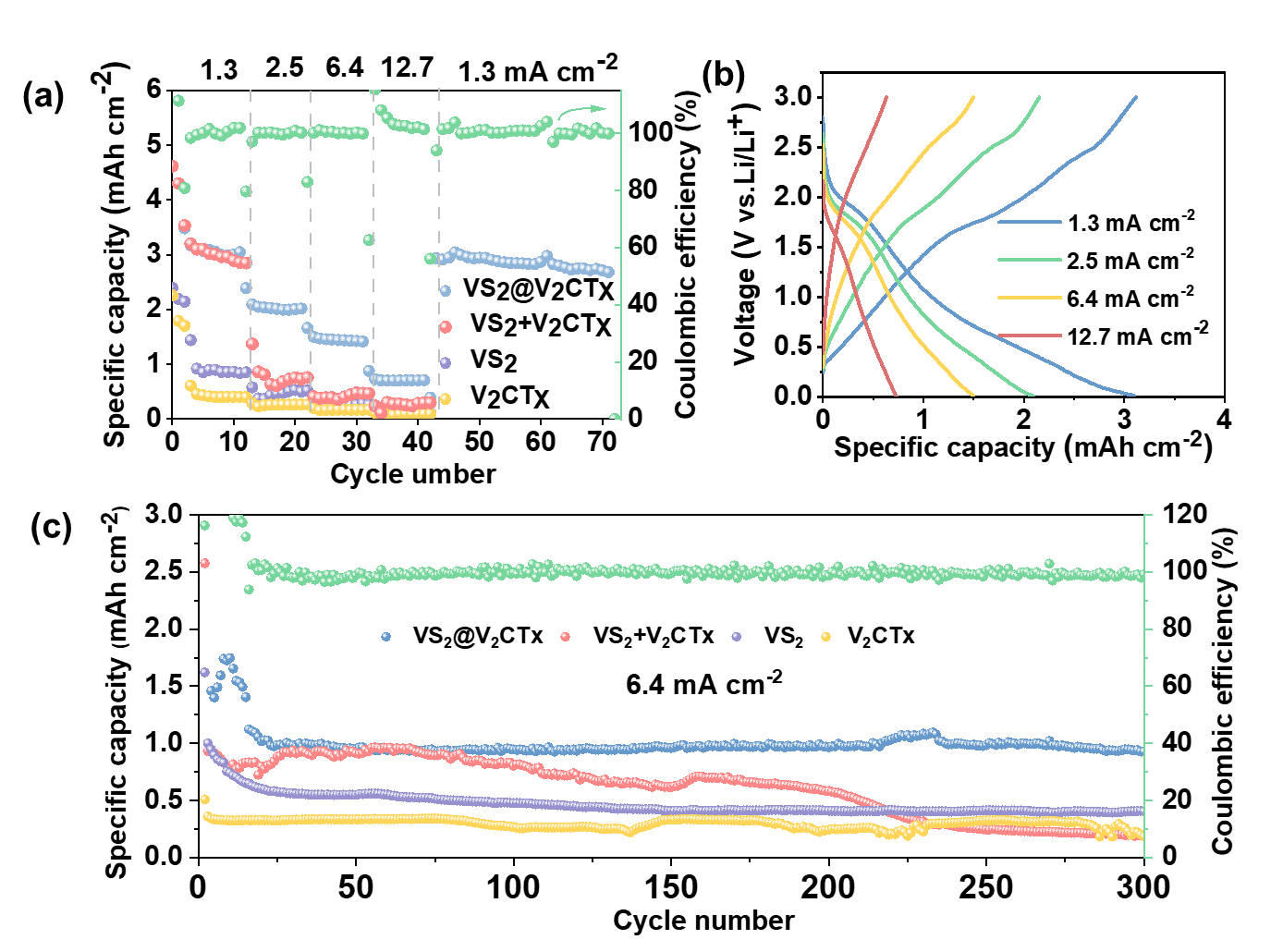


Supplementary Figure 12. (a) Rate capacity performance is measured at different current. (b) Galvanostatic discharge and charge profiles of VS_2_@V_2_CT_X_ were measured at the different current. (c) Cycling performance was measured at current densities of 6.4 mA cm^-2^.


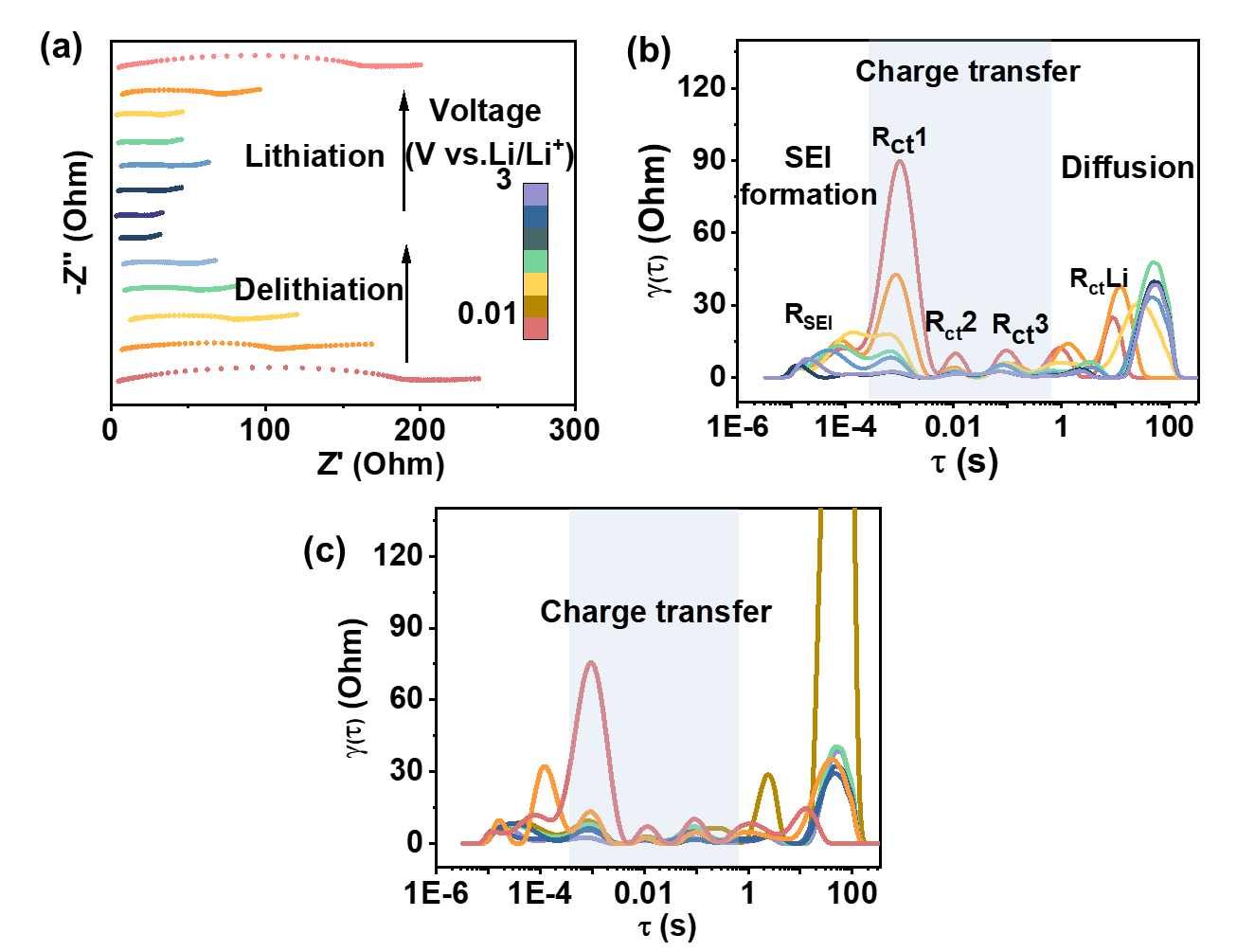


Supplementary Figure 13. (a) EIS evolution at the lithiation and delithiatiion of VS_2_@V_2_CT_X_ after the first discharge is completed. (b) DRT transition of EIS in (a). (c) DRT transition of EIS during charge process from 0.01 to 3 V.

Supplementary Figure 14. measurements feature the 1^st^ and 2^nd^ cycle of VS_2_@V_2_CT_X_ under 0.2 mV s^−1^ in the voltage window between 0.01–3 V.

Supplementary Figure 15, XPS analysis of S element inVS_2_@V_2_CT_X_ at different charge/discharge stage.


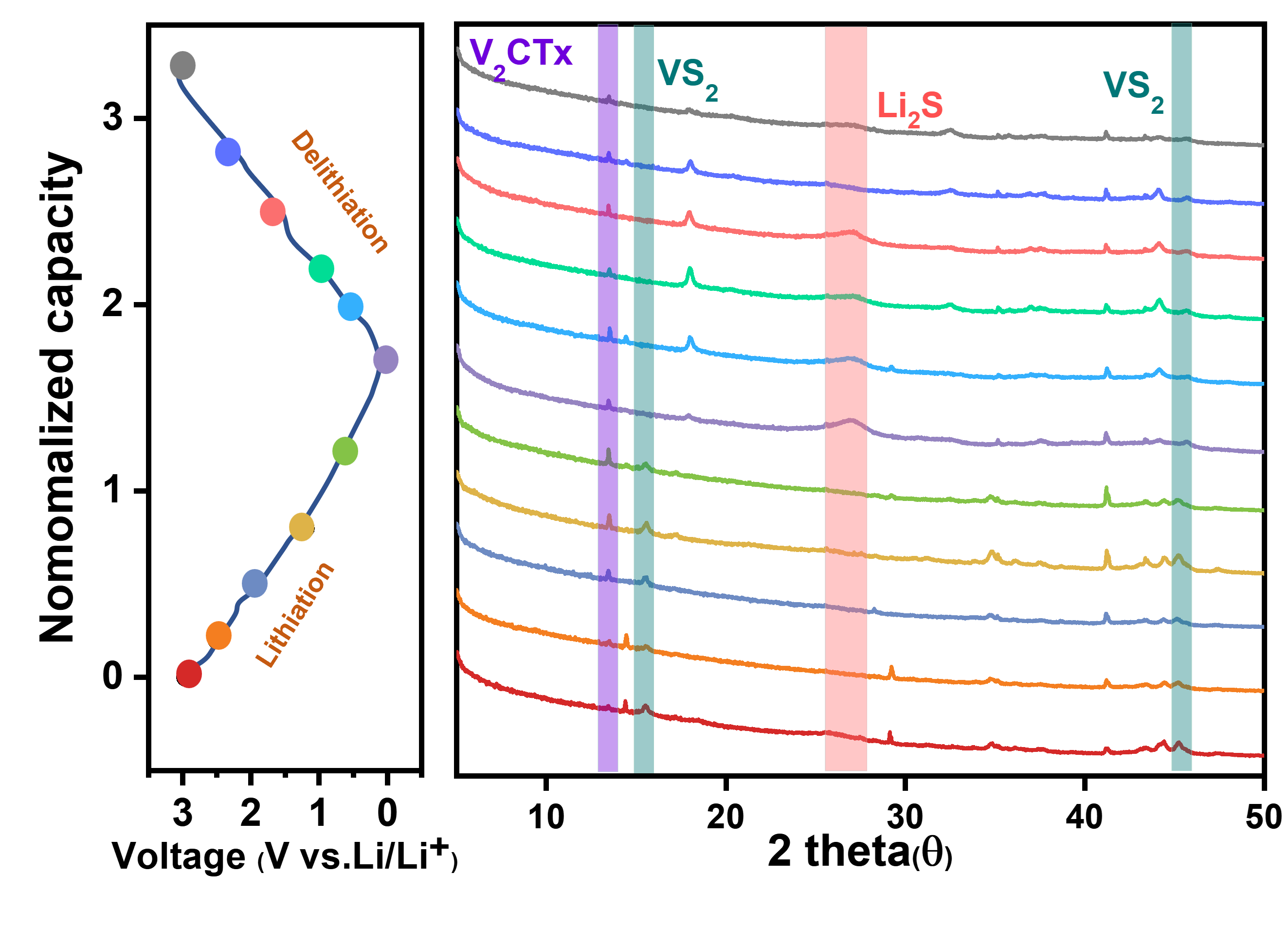


Supplementary Figure 16. Ex situ XRD patterns of VS_2_@V_2_CT_X_ anode during the first discharge–charge stages.


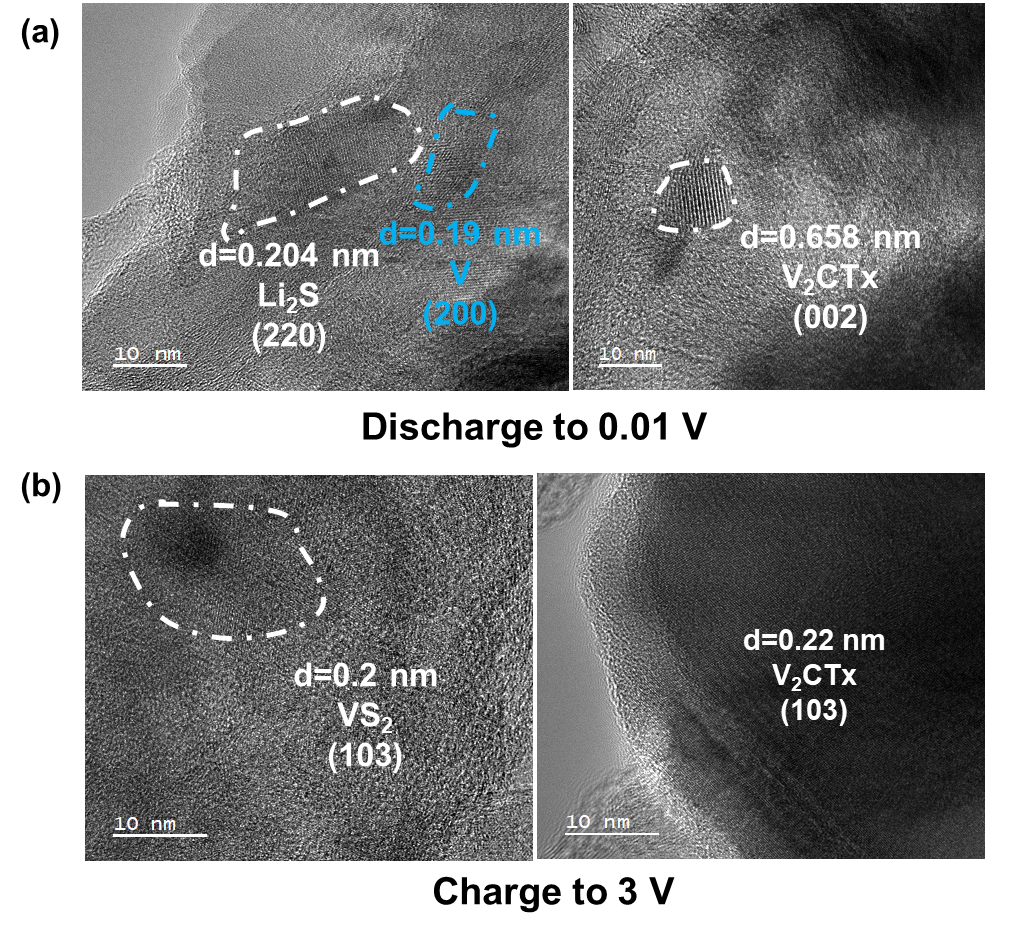


Supplementary Figure 17.TEM images of VS_2_@V_2_CT_X_ at different charge/discharge stage.


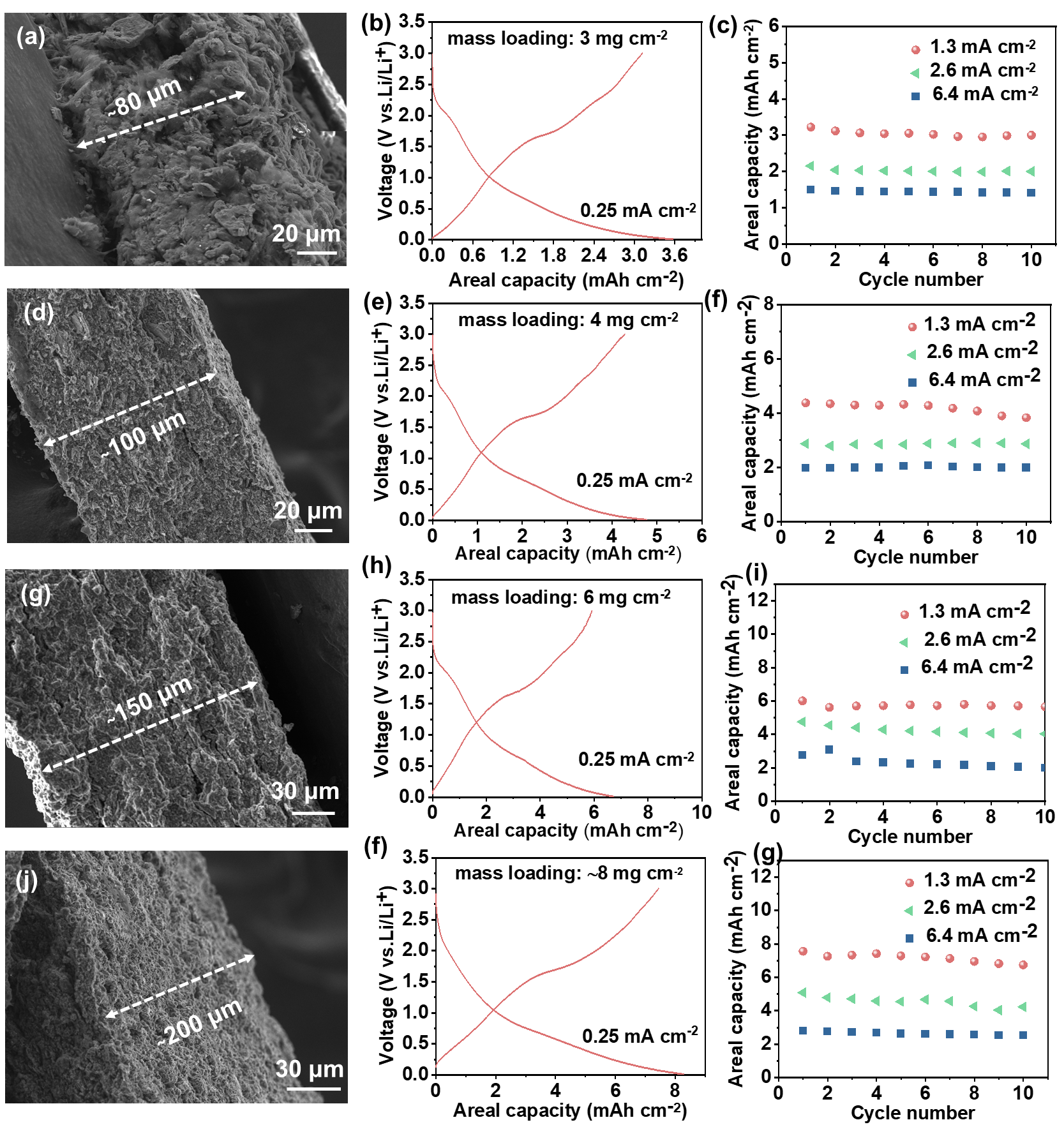


Supplementary Figure 18. Cross-sectional SEM images, galvanostatic charge–discharge curves and Cycling performance under different current density of the VS_2_@V_2_CTx anode with 80, 100, 150 and 200 μm.


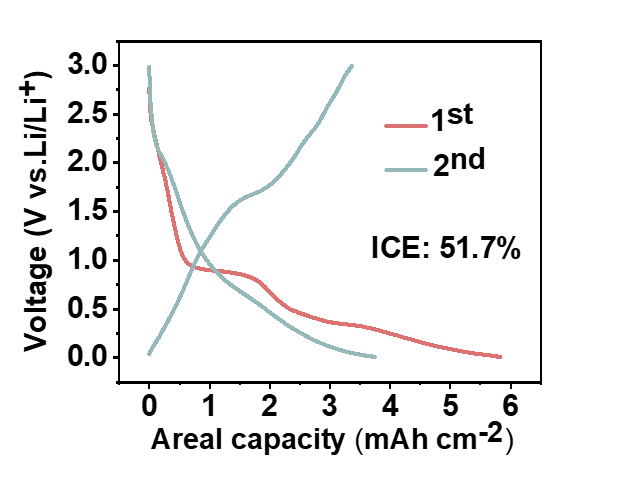


Supplementary Figure 19. The Initial Coulombic Efficiency (ICE) of VS_2_@V_2_CTx electrode with the thickness of 100 μm at 0.25 mA cm^-2^.


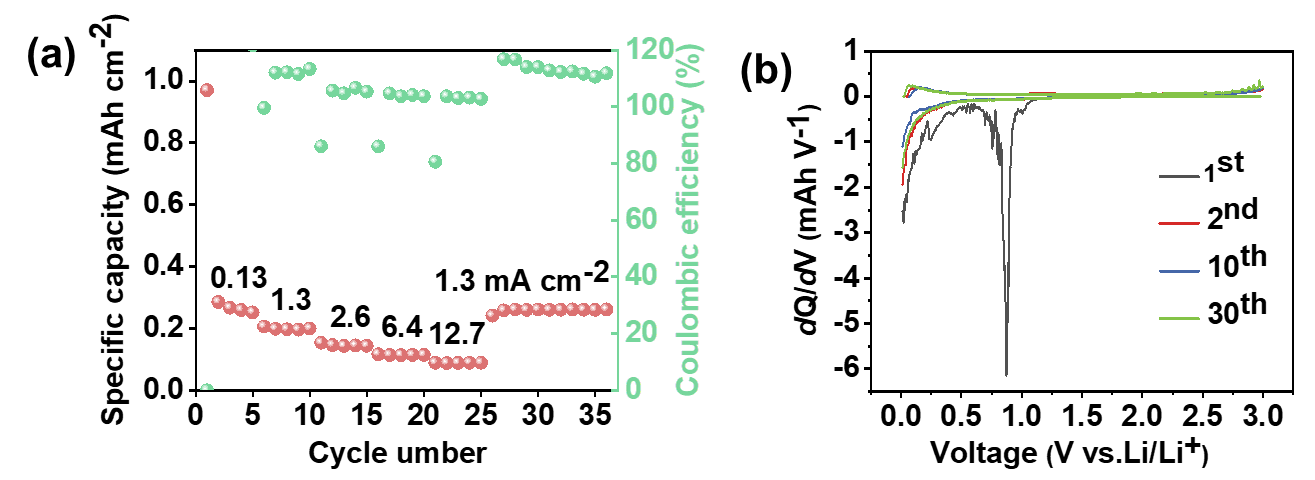


Supplementary Figure 20.（a）Rate performance of pure MWCNTs. (b) Electrochemical performance of pure MWCNTs current collector. Differential capacity curves of the MWCNts at different cycle with 1.3 mA cm^-2^, explanation MWCNTs does not provide capacity after the first discharge.


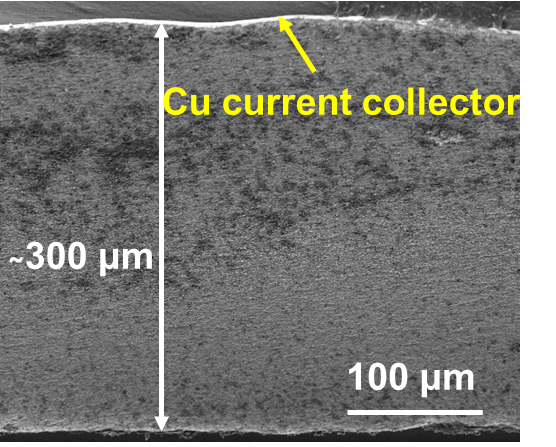


Supplementary Figure 21. Traditional graphite electrode prepared by slurry coating, 300 μm thick.


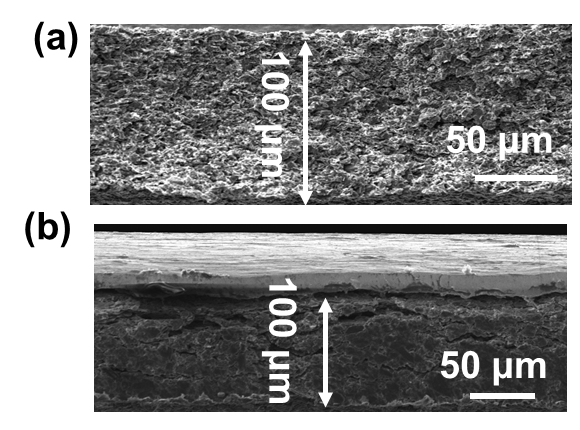


Supplementary Figure 22. Cross-sectional SEM images of the VS_2_@V_2_CTx (a) and traditional graphite electrode (b) after thinning to 100 μm.

**Supplementary Table 1. Li^+^ content at a depth of 200 μm for different electrodes**

| **Point** | **Peak area** | | **Relative content (%)** | |
| --- | --- | --- | --- | --- |
|  | **VS_2_@V_2_CTx electrode** | **Graphite electrode** | **VS_2_@V_2_CTx electrode** | **Graphite electrode** |
| 1 | 7579 | 454 | 100 | 7.2 |
| 2 | 5828 | 477 | 74.3 | 6.3 |
| 3 | 4594 | 160 | 60.6 | 2.1 |
| 4 | 6247 | 285 | 82.4 | 3.8 |
| 5 | 5940 | 398 | 78.4 | 5.3 |
| Note: the relative content is based on the value 7579. | | | | |


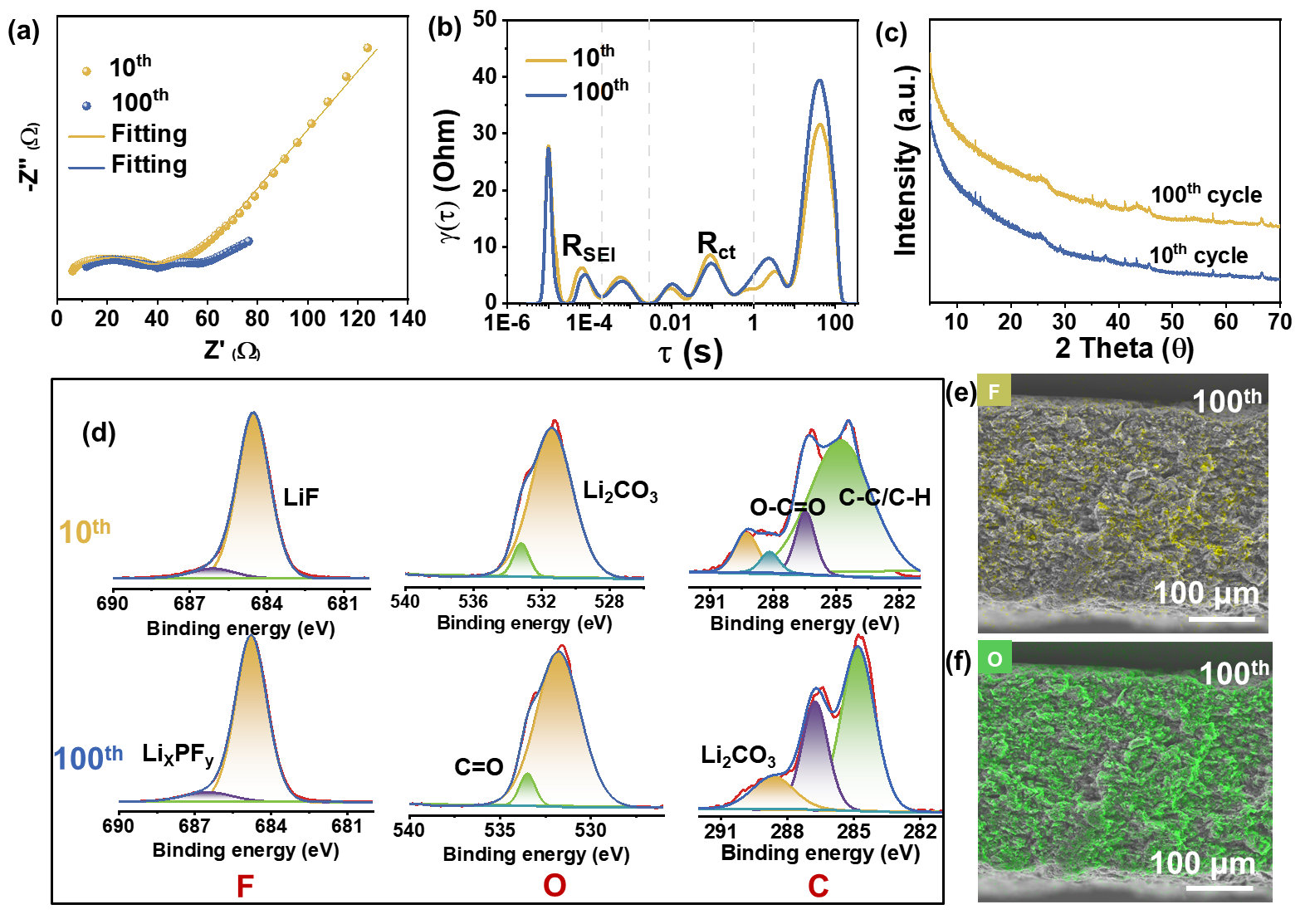


Supplementary Figure 23. (a) Nyquist plots of VS_2_@V_2_CT_X_ electrode at a fully discharged state after ten and one hundred cycles at 6.4 mA cm^-2^. (b) DRT transition result based on EIS. (c) Composition characterization of VS_2_@V_2_CT_X_ electrodes by XRD after different cycle numbers prove the SEI layer composition almost unchanged. (d) XPS spectra of the SEI formed on VS_2_@V_2_CTx electrodes with different cycle numbers (10^th^ and 100^th^). (e) Cross-sectional SEM images and corresponding EDS elemental maps of the VS_2_@V_2_CTx after 100 cycles.


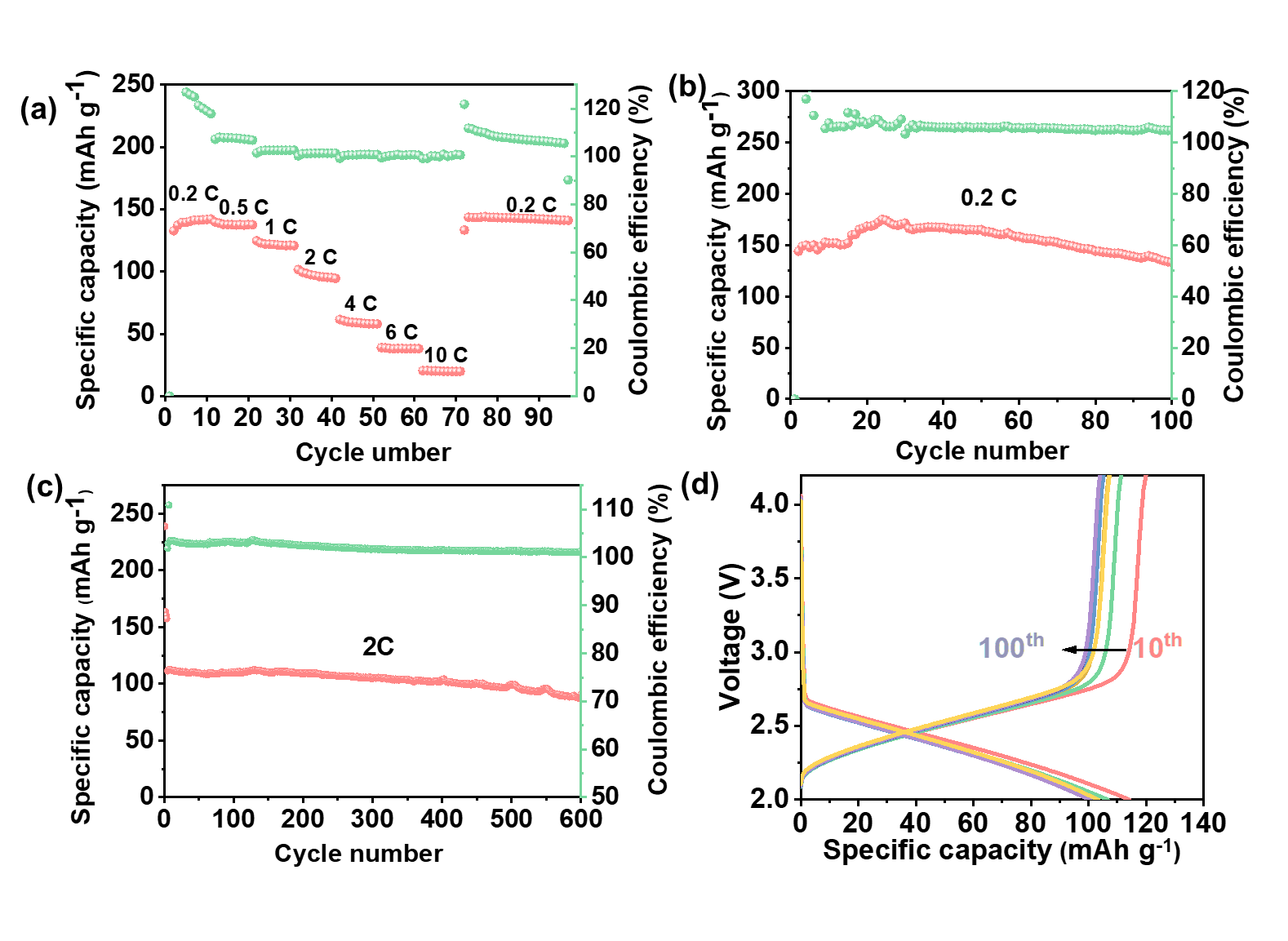


Supplementary Figure 24. (a) rate performance. (b-c) Specific capacity and coulombic efficiency measured at the current density of 0.2C and 2C, respectively. The specific capacity of the full cell is calculated based on the mass of the LiFePO_4_ anode electrode. (d) Voltage profiles are plotted as a function of cycles at a discharge rate of 2C.


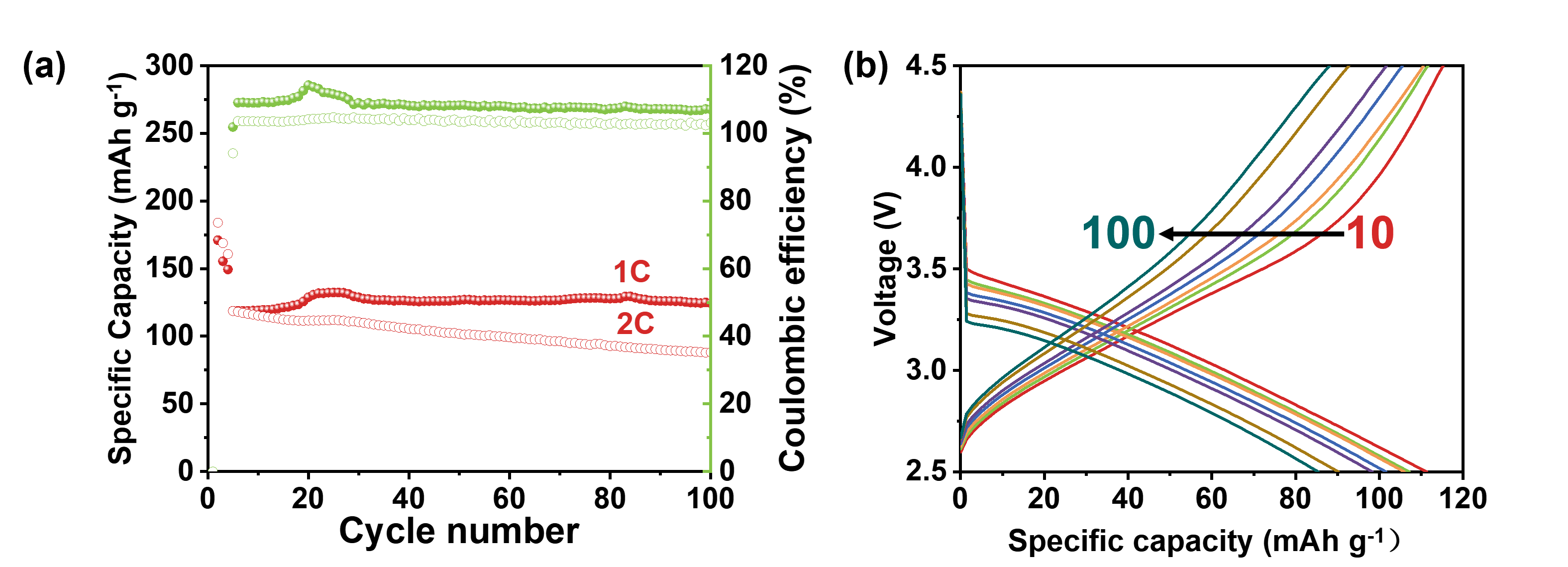


Supplementary Figure 25. Electrochemical performance of VS_2_@V_2_CT_X_||NCM811 full cell. (a) Specific capacity and Coulombic efficiency measured at the current density of 1C and 2C (1C=180 mA g^-1^), respectively. (b) Voltage profiles plotted as a function of the current density in the voltage range of 2.6-4.5

**Supplementary Table 2. Summary of some other reported Li-ion battery anodes performance in comparison with our research**

| **Number** | **Anode materials** | **Active loading (mg cm^-2^)** | **Electrode thickness**  **(μm)** | **Areal capacity (mAh cm^-2^)** | **Areal current density (mA cm^-2^)** | **Published journal** |
| --- | --- | --- | --- | --- | --- | --- |
|  | VS_2_@V_2_CTx | 13 | 300 | 13.6 | 0.25 | / |
|  |  |  |  | 10 | 1.3 |  |
|  |  |  |  | 8 | 2.6 |  |
|  |  |  |  | 2.3 | 6.4 |  |
| 1 | MoSe_2_/MXene | 1.5-1.8 | 15 | 0.485 | 0.89 | Journal of Power Sources^[3]^ |
| 2 | phosphorene/Ti_3_C_2_Tx | 1.5-2.0 | 20 | ~1 | 1.8 | Nano-Micro Letters^[4]^ |
| 3 | Li_4_Ti_5_O_12_/Ti_3_C_2_CTx | 7 | 65 | 0.54 | 0.62 | Energy Storage Materials^[5]^ |
| 4 | nanocellulose-carbon-nanotube (NCN)/ LiCoO_2_ | 20 | 400 | 2.1 | 0.5 | Energy Environmental Materials^[6]^ |
| 5 | Prussian Blue Analogues (PBA/Ti_3_C_2_Tx) | 0.8-0.91 | 13 | 1.37 | 0.18 | Advanced materials^[7]^ |

**References**

[1] aC. X. Chen, X.; Anasori, B.; Sarycheva, A.; Makaryan, T.; Zhao, M.; Urbankowski, P.; Miao, L.; Jiang, J.; Gogotsi, Y.. , *Angew. Chem., Int. Ed* **2018**, *57*, 1846–1850; bC. S. Ling, L.; Ouyang, Y.; Chen, Q.; Wang, J., *Advanced Science* **2016**, *3*, 1600180.

[2] Y. X. Liang Zhao, Xiaoxuan Wang, Rui Zhao, Xinyue Chi, Yixiang Zhou, Huaizhi Wang, Zhiyu Yang, Yi-Ming Yan, *Small* **2022**, *18*.

[3] P. Y. Fuxing Yin, Wenjing Yuan, Alexander Semencha, Chengwei Zhang, Puguang Ji, Gongkai Wang,, *Journal of Power Sources* **2021**, *488*, 229452.

[4] J. X. Yihui Li, Ruofei Wang, Shugang Min, *Nano-Micro Letters* **2024**, *16*.

[5] L. Feng, S. Zhou, H. Cui, R. A. Soomro, P. Zhang, B. Xu, *Energy Storage Materials* **2025**, *75*, 104079.

[6] Z. Du, Q. Zha, Z. Zhang, Q. Chen, H. Yang, Z. Lu, T. Zhai, H. Li, *ENERGY & ENVIRONMENTAL MATERIALS* **2025**, *8*, e12867.

[7] Y. Shi, G. Song, B. Yang, Y. Tang, Z. Liu, Z. Zhang, M. Shakouri, J. Cheng, H. Pang, *Advanced Materials* **2025**, *37*, 2416665.
